# Proteomics‐Empowered Microfluidic‐SERS Immunoassay for Identifying and Detecting Biomarkers of Micropapillary Lung Adenocarcinoma

**DOI:** 10.1002/advs.202501336

**Published:** 2025-05-03

**Authors:** Dechun Zhang, Kaiming Peng, Hui Xu, Yanping Chen, Jing Wang

**Affiliations:** ^1^ Key Laboratory of Optoelectronic Science and Technology for Medicine of Ministry of Education Fujian Provincial Key Laboratory of Photonics Technology Fujian Normal University Fuzhou Fujian 350117 China; ^2^ Department of Thoracic Surgery Fujian Medical University Union Hospital Fuzhou Fujian 350001 China; ^3^ Department of Pathology Clinical Oncology School of Fujian Medical University and Fujian Cancer Hospital Fuzhou Fujian 350014 China

**Keywords:** biomarker discovery, micropapillary lung adenocarcinoma, proteomics, surface‐enhanced raman scattering

## Abstract

The presence of a micropapillary (MPP) component is a crucial determinant of surgical strategies for lung adenocarcinoma (LUAD), yet reliable blood biomarkers for predicting MPP⁺ LUAD remain elusive. Here, we integrate 4D label‐free quantitative proteomics, a nanomixing‐enhanced microfluidic surface‐enhanced Raman spectroscopy (SERS) platform, and machine learning to sensitively identify and validate blood protein biomarkers associated with MPP⁺ LUAD. Comparative proteomics reveal 44 differentially expressed proteins (DEPs) between MPP⁺ and MPP⁻ LUADs, with bioinformatics uncovering their roles in MPP⁺ LUAD formation. To enable sensitive, multiplex detection of 4 upregulated DEPs, the nanomixing effect is leveraged to enhance target protein‐SERS barcode interactions while minimizing nonspecific binding to antibody‐functionalized gold electrodes. The SERS barcode cocktail allows simultaneous detection of the 4 selected DEPs. Machine learning models based on SERS detection effectively distinguish MPP⁺ from MPP⁻ LUAD patients, as well as LUAD patients from healthy donors. This approach demonstrates strong diagnostic potential for early, non‐invasive MPP detection in LUAD, advancing nanotechnology‐driven disease diagnosis and monitoring.

## Graphical abstract

The presence of a micropapillary (MPP) component is critical for lung adenocarcinoma (LUAD) surgery, yet reliable blood biomarkers remain lacking. This study integrates proteomics for biomarker identification, a nanomixing‐enhanced surface‐enhanced Raman spectroscopy (SERS) platform for sensitive detection, and machine learning for accurate differentiation of MPP⁺ from MPP⁻ LUAD and LUAD from healthy donors, advancing non‐invasive diagnostics.

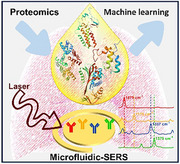

## 1 Introduction

Lung cancer remains the leading cause of cancer‐related mortality worldwide, with adenocarcinoma accounting for nearly half of all cases.^[^ ^1^, ^2^ ^]^ This malignancy exhibits considerable histological heterogeneity, with distinct clinicopathologic and genomic characteristics across subtypes. Surgical resection, particularly curative‐intent surgery, is the primary treatment for early‐stage lung adenocarcinoma (LUAD); however, postoperative relapse occurs in 30%–60% of cases.^[^ ^3^ ^]^ Among LUAD subtypes, the micropapillary pattern (MPP) is of particular clinical concern due to its strong association with recurrence and metastasis, even when comprising less than 5% of the tumor.^[^ ^4^, ^5^ ^]^ Consequently, accurate preoperative identification of MPP⁺ LUAD is critical for guiding surgical decisions and improving patient outcomes.

Current preoperative assessments for MPP⁺ LUAD rely primarily on histological puncture biopsy, which is invasive and samples only a small portion of the tumor, limiting its ability to capture tumor heterogeneity. Despite the clinical significance of MPP⁺ LUAD, no reliable preoperative biomarkers or methods exist for its accurate diagnosis. Proteomic approaches, particularly mass spectrometry‐based proteomics, enable comprehensive profiling of protein expression and offer insights into tumor biology.^[^ ^6^ ^]^ Several studies have applied label‐free quantitative proteomics to map the proteomic landscape of LUAD. For instance, Huang et al. characterized proteomic differences between early‐stage LUAD and benign lung diseases.^[^ ^7^ ^]^ Zhou et al. identified differentially expressed proteins (DEPs) between low‐risk and high‐risk (MPP or solid predominant) early‐stage LUAD subtypes.^[^ ^8^ ^]^ However, despite these advancements, blood biomarkers capable of predicting MPP⁺ LUAD remain elusive.

Surface‐enhanced Raman scattering (SERS) has gained increasing attention in biomedical research due to its ability to provide molecular structural information with ultra‐sensitivity, multiplexing capability, and photostability.^[^ ^9^ ^]^ SERS has been explored for disease diagnostics through two main strategies: i) direct SERS measurement of clinical samples combined with machine learning for disease differentiation^[^ ^10^ ^]^ and ii) SERS‐based probing systems for targeted biomarker detection.^[^ ^11^, ^12^, ^13^ ^]^ While promising diagnostic accuracies have been achieved, detecting diseases with elusive biomarkers remains a significant challenge. This is partly due to the predominant focus on developing new detection strategies rather than integrating biomarker discovery with SERS sensing platforms.

To address this gap, we propose the first roadmap from cancer‐associated protein biomarker discovery to the development of a SERS‐based diagnostic platform. We employed 4D label‐free quantitative proteomics, integrating liquid chromatography, mass‐to‐charge ratio analysis, retention time, and ion mobility separation, to identify DEPs in serum samples from MPP⁺ and MPP⁻ LUAD patients. We then developed a nanomixing‐enhanced microfluidic‐SERS platform for the sensitive, multiplex detection of DEPs significantly upregulated in MPP⁺ LUAD. Finally, machine learning algorithms were applied to assess the SERS‐based approach in distinguishing MPP⁺ from MPP⁻ LUAD patients and LUAD patients from healthy donors. This study pioneers a biomarker discovery‐integrated SERS nanotechnology approach, establishing a proteomics‐driven SERS diagnostic platform that seamlessly links biomarker discovery with the development of sensing platforms for disease diagnostics.

## 2 Results and Discussion

### 2.1 Working Scheme

**Figure 1:**
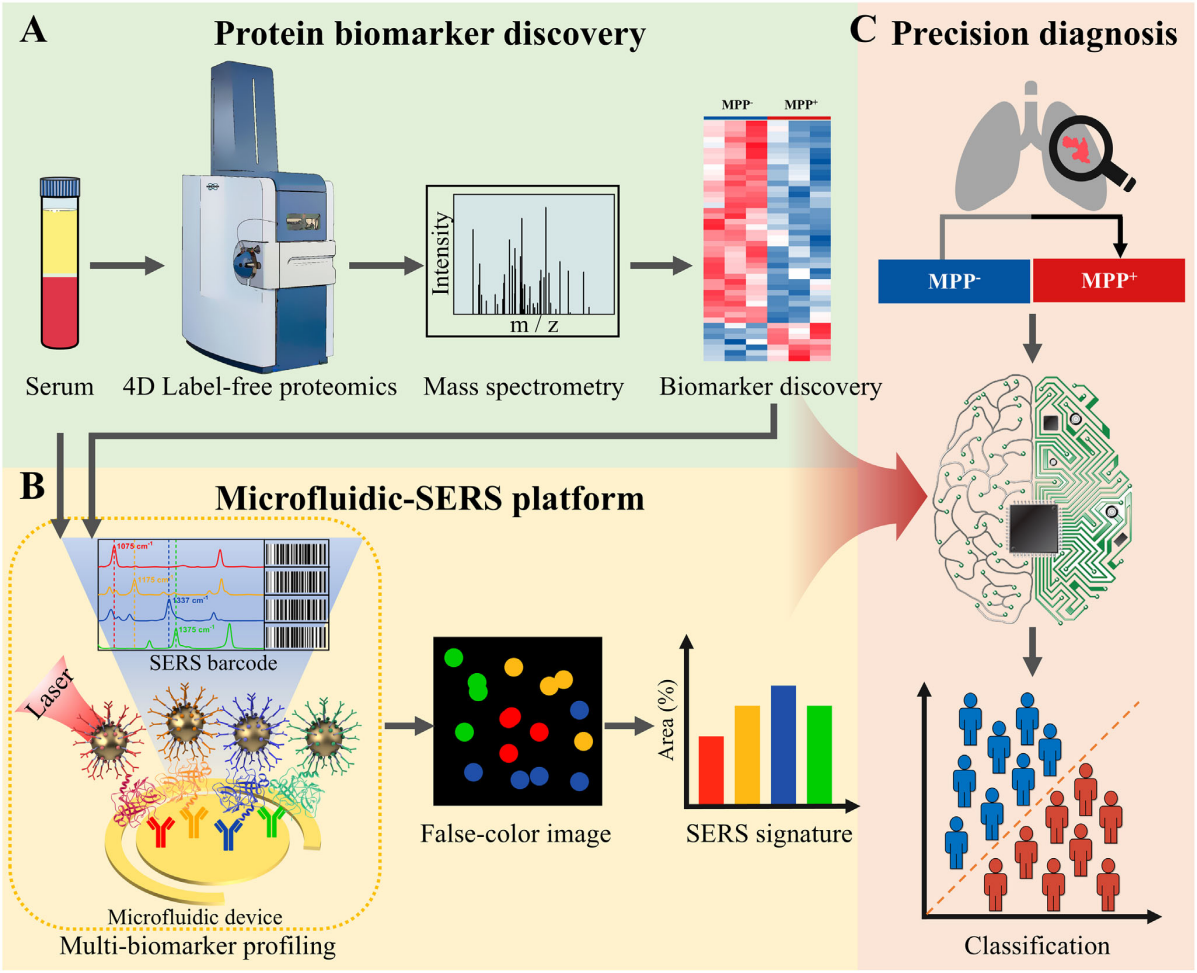
Workflow from blood biomarker discovery to the development of a microfluidic‐SERS immunoassay for MPP⁺ LUAD detection. A) 4D label‐free quantitative proteomics was employed to identify blood protein biomarkers associated with MPP⁺ LUAD. B) A microfluidic‐SERS platform was developed for the multiplex detection of these biomarkers. Gold electrodes were functionalized with capture antibodies to enrich target blood proteins, followed by labeling with SERS barcodes. The SERS barcodes, consisting of gold nanoparticles (AuNPs conjugated with antibodies and Raman reporters, generated distinct Raman spectra upon laser excitation. For each biomarker, abundance was quantified by measuring the area of barcode signal‐present pixels, and the combined expression profile for each patient sample was presented as a “SERS signature”. C) Machine learning algorithms were then applied to accurately identify LUAD patients and to differentiate MPP⁺ from MPP^−^ LUAD patients.

**Figure** **1** illustrates the working scheme and workflow for identifying protein biomarkers of MPP⁺ LUAD using our 4D label‐free proteomics‐assisted microfluidic‐SERS platform. Blood serves as an ideal source for tumor biomarker discovery due to its minimally invasive collection and comprehensive representation of tumor biology across lesion sites. To identify DEPs between MPP⁺ and MPP⁻ LUAD patients, 4D label‐free quantitative proteomic analysis was first performed on serum samples, enabling unbiased and sensitive proteomic profiling (Figure 1A). The identified DEPs were then subjected to bioinformatics analysis to explore their molecular functions and biological involvement.

To validate the DEPs, a nanomixing‐enhanced microfluidic‐SERS immunoassay was performed (Figure 1B). The microfluidic chip featured arrays of asymmetric electrodes, consisting of inner circular and outer ring structures. Applying an alternating current electric field to these electrodes generated a nanomixing effect that enhanced mass transfer and interfacial collisions, improving reaction efficiency. Gold electrodes functionalized with capture antibodies enabled target‐specific protein binding, while SERS barcodes facilitated simultaneous detection of 4 protein candidates. Upon laser scanning, the barcode cocktail emitted distinct “spectral barcodes”, allowing multiplexed protein detection. A false‐color SERS spectral image was generated based on the characteristic peaks of each barcode, reflecting the relative abundance of target proteins in blood samples. The active pixel area for each protein candidate was calculated to generate the “SERS signature”. These SERS signatures were then used as input for machine learning analysis to assess the clinical utility of the nanomixing‐enhanced microfluidic‐SERS immunoassay in identifying LUAD patients and distinguishing MPP⁺ from MPP⁻ LUAD patients.

In comparison to our previous work and other SERS‐related biosensing studies, this work introduces two key innovations: i) we present a novel approach that combines proteomics‐driven biomarker discovery with SERS technology, demonstrating its potential to improve disease detection, particularly for conditions with elusive biomarkers; ii) this study marks the first attempt to apply SERS technology for detecting MPP⁺ LUAD, a subtype where early detection is critical for guiding treatment strategies and currently depends on invasive biopsy techniques. These contributions underscore the unique impact of our work.

### 2.2 Proteomic and Bioinformatic Analyses Reveal Altered Blood Protein Profiles in MPP⁺ LUAD

**Figure 2:**
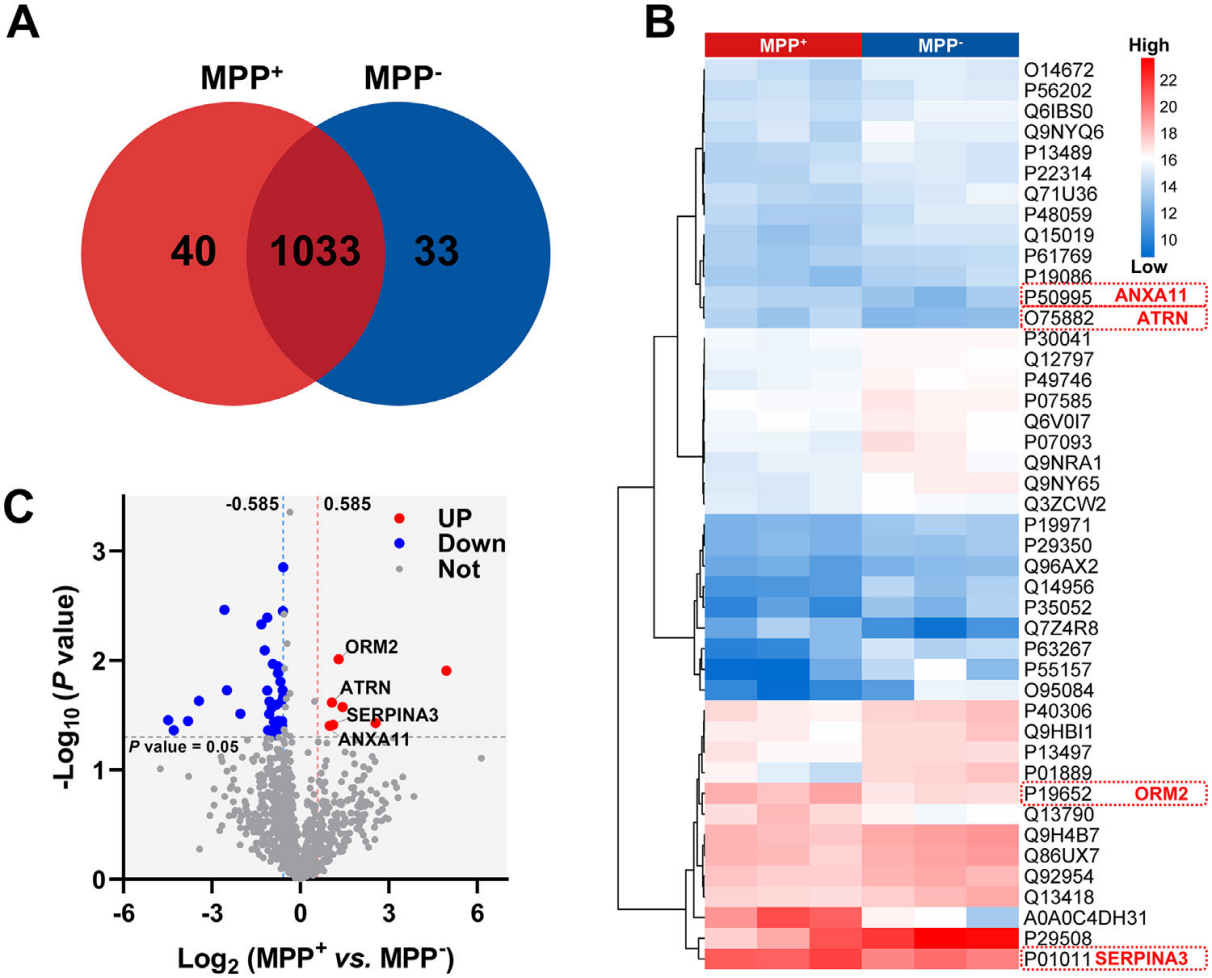
Serum protein levels analyzed by 4D label‐free quantitative proteomics. A) Venn diagram illustrating shared and unique serum proteins in MPP⁺ (P1‐P3) and MPP⁻ (P19‐P21) LUAD patients. B) Heat map displaying enrichment of 44 DEPs, clustered based on abundance patterns. C) Volcano plot highlighting DEPs between MPP⁺ and MPP⁻ LUAD patients, with upregulated proteins in red, downregulated in blue, and non‐significant in gray (fold change > 1.5, *P* < 0.05).

We employed 4D label‐free quantitative proteomics to analyze serum samples from MPP⁺ and MPP⁻ LUAD patients, identifying 1106 proteins across six patient samples. Of these, 93.4% (1033) were shared between both groups (**Figure** **2**A). Proteins with a fold change > 1.5 and a *P*‐value < 0.05 were classified as DEPs. Clustered heat maps revealed variations in DEP enrichment across individual patients (Figure 2B). The volcano plot (Figure 2C) and Table S1 (Supporting Information) summarize 44 DEPs, including 7 upregulated and 37 downregulated proteins. These findings highlight significant proteomic differences between MPP⁺ and MPP⁻ LUAD patients.

**Figure 3:**
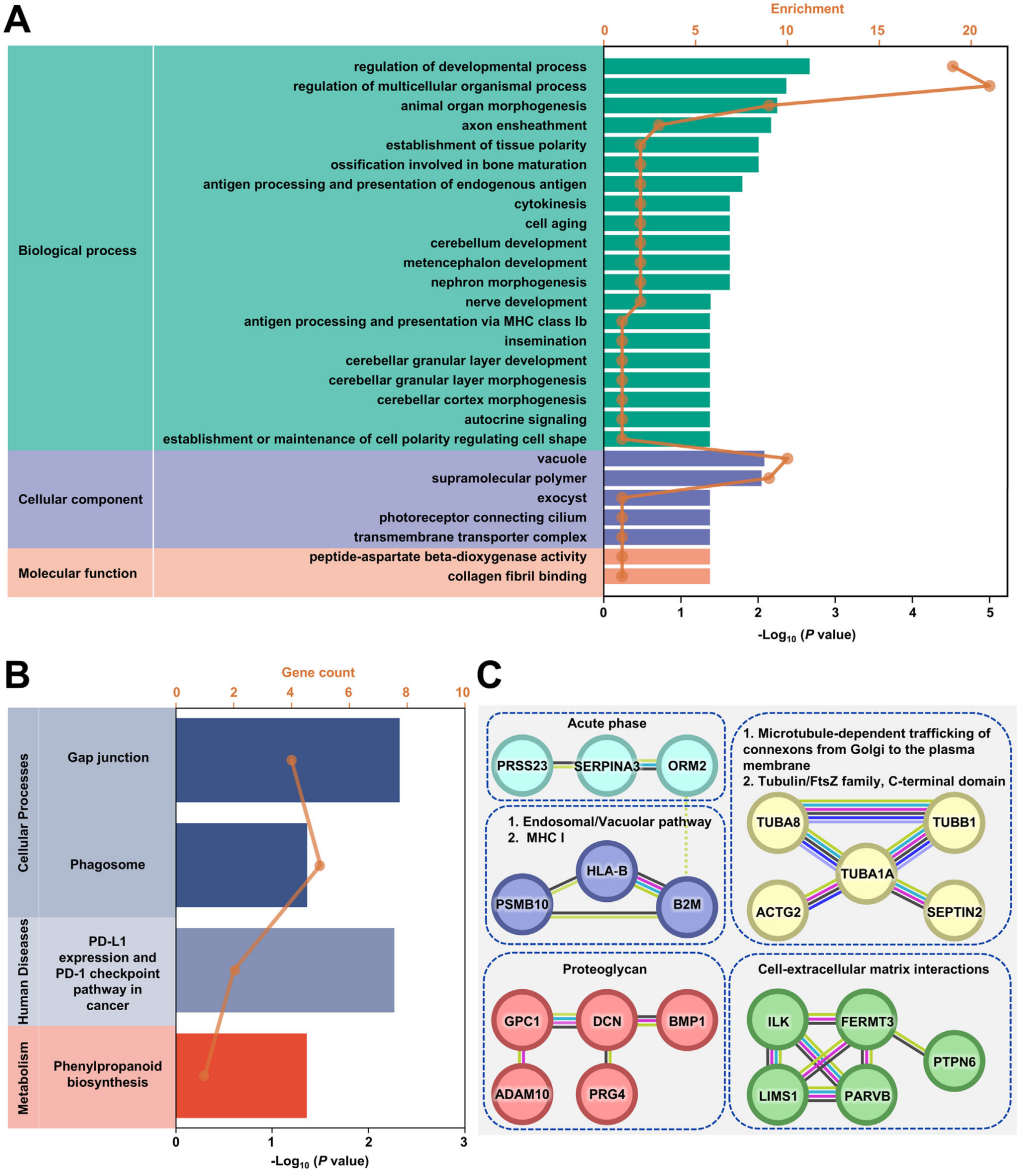
Bioinformatic analyses of DEPs between MPP⁺ and MPP⁻ LUAD patients, including A) GO annotation, B) KEGG pathway enrichment, and C) PPI network analysis.

To investigate the functional significance of these DEPs, we performed Gene Ontology (GO) annotation analysis (**Figure** **3**A). DEPs were categorized based on biological processes, cellular components, and molecular functions. Most were enriched in biological processes related to developmental regulation and multicellular organismal processes. For cellular components, DEPs were significantly associated with the vacuole. Kyoto Encyclopedia of Genes and Genomes (KEGG) pathway analysis revealed that 27 downregulated DEPs were involved in pathways such as phagosome formation, gap junctions, PD‐L1/PD‐1 checkpoint signaling in cancer, and phenylpropanoid biosynthesis (Figure 3B). These pathway enrichments suggest that DEPs play key roles in immune regulation and tumor microenvironment interactions in MPP⁺ LUAD.

To explore DEP interactions, we constructed a protein‐protein interaction (PPI) network using the STRING database (Figure 3C). Each node represents a protein, and each edge denotes an interaction. Of the 44 DEPs, 21 exhibited known interactions, while 23 showed no associations in STRING v12. Network analysis identified two upregulated acute‐phase proteins, SERPINA3 and ORM2, in MPP⁺ LUAD patients. Wang et al. previously reported that longitudinal changes in acute‐phase proteins (e.g., ORM2, SERPINA1) correlate with treatment response and tumor progression.^[^ ^14^ ^]^ Additionally, 19 downregulated DEPs were associated with pathways such as proteoglycan signaling, microtubule‐dependent trafficking of connexons, tubulin/FtsZ family functions, cell‐extracellular matrix interactions, the endosomal/vacuolar pathway, and MHC I antigen presentation. These interactions underscore the involvement of DEPs in tumor‐associated inflammatory responses and cellular communication pathways.

Based on proteomic analysis (Figure 2C) and antibody availability, we selected 4 upregulated DEPs—ANXA11, ORM2, SERPINA3, and ATRN—for further investigation. ANXA11 dysfunction has been linked to drug resistance and metastasis in multiple cancers.^[^ ^15^ ^]^ Immune‐related serum biomarkers, such as ORM1 and ORM2, have been proposed for early non‐small cell lung cancer diagnosis.^[^ ^16^, ^17^ ^]^ SERPINA3, a serine protease inhibitor, plays a role in the acute‐phase response, inflammation, and proteolysis. Its dysregulation and altered glycosylation have been associated with tumor progression and recurrence, making it a potential biomarker for tumor monitoring.^[^ ^18^, ^19^, ^20^ ^]^ Wang et al. developed a plasma‐based prognostic score for ALK‐positive non‐small cell lung cancer patients using the baseline expression levels of 5 plasma proteins (SERPINA4, ATRN, APOA4, TF, and MYOC).^[^ ^14^ ^]^ Collectively, these selected DEPs represent promising candidates for further validation and clinical application in MPP⁺ LUAD detection.

### 2.3 Characterization of SERS Barcodes

**Figure 4:**
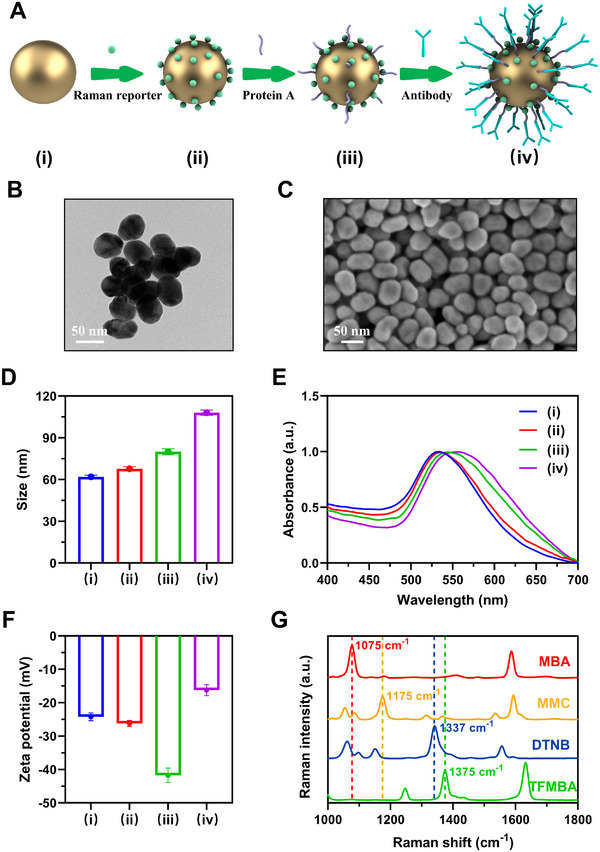
Characterization of SERS barcodes. A) Schematic illustration of the SERS barcode synthesis process: (i) AuNPs; (ii) AuNPs conjugated with Raman reporters (Ra) and DSP; (iii) protein A‐functionalized AuNPs@Ra+DSP; (iv) antibody‐conjugated AuNPs@Ra+DSP@protein A. B) Transmission electron microscopy and C) scanning electron microscopy images of AuNPs. D) Size distribution analysis, E) UV–vis spectra, and F) zeta potential measurements of synthesized particles at different stages. G) Raman spectra of SERS barcodes labeled with MBA, MMC, DTNB, or TFMBA. Error bars in Figure 4D,F represent the standard error of the mean (SEM) from three independent experiments.

To enable the simultaneous detection of multiple target proteins, we synthesized and characterized 4‐plex SERS barcodes. The synthesis process is illustrated in **Figure** **4**A. Raman reporters and dithiobis(succinimidyl propionate) (DSP) protein linkers were covalently conjugated to the surface of AuNPs via Au─S bonds. Protein A was subsequently attached through the reaction of its amino groups with the N‐hydroxysuccinimide (NHS) ester of DSP, forming stable amide bonds. Due to its high affinity for the Fc region of IgG antibodies, protein A facilitated the oriented conjugation of antibodies onto AuNPs, ensuring optimal antigen‐binding accessibility.

Comprehensive characterization confirmed the successful synthesis of SERS barcodes. Transmission electron microscopy and scanning electron microscopy images revealed that AuNPs had an average size of ≈60 nm (Figure 4B,C). Hydrodynamic size analysis showed an increase following protein A and antibody conjugation (Figure 4D). The UV–vis spectra of AuNPs and AuNPs conjugated with Raman reporters and DSP (AuNPs@Ra+DSP) exhibited localized surface plasmon resonance (LSPR) bands at ≈540 nm (Figure 4E). A red shift was observed after protein A attachment, indicating an increase in refractive index, with an additional shift following antibody conjugation. As shown in Figure 4F, after antibody conjugation, the zeta potential of protein A‐modified AuNPs increased from −41.71 ± 2.16 to −16.25 ± 1.66 mV, due to the positively charged lysine residues in antibodies at pH 7, demonstrating successful antibody conjugation. Similar phenomena have been reported in previous studies.^[^ ^21^, ^22^ ^]^ Raman spectra of the synthesized SERS barcodes, conjugated with different Raman reporter‐antibody pairs, displayed distinct characteristic peaks: 1075 cm⁻¹ (4‐Mercaptobenzoic acid, MBA), 1175 cm⁻¹ (2,7‐mercapto‐4‐methylcoumarin, MMC), 1337 cm⁻¹ (5,5′‐dithiobis(2‐nitrobenzoic acid), DTNB), and 1375 cm⁻¹ (2,3,5,6‐tetrafluoro‐MBA, TFMBA) (Figure 4G). These results confirm the successful synthesis of SERS barcodes with covalent Raman reporter conjugation and oriented antibody immobilization, ensuring high specificity and signal reproducibility.

### 2.4 Evaluation of the Microfluidic‐SERS Platform Performance

**Figure 5:**
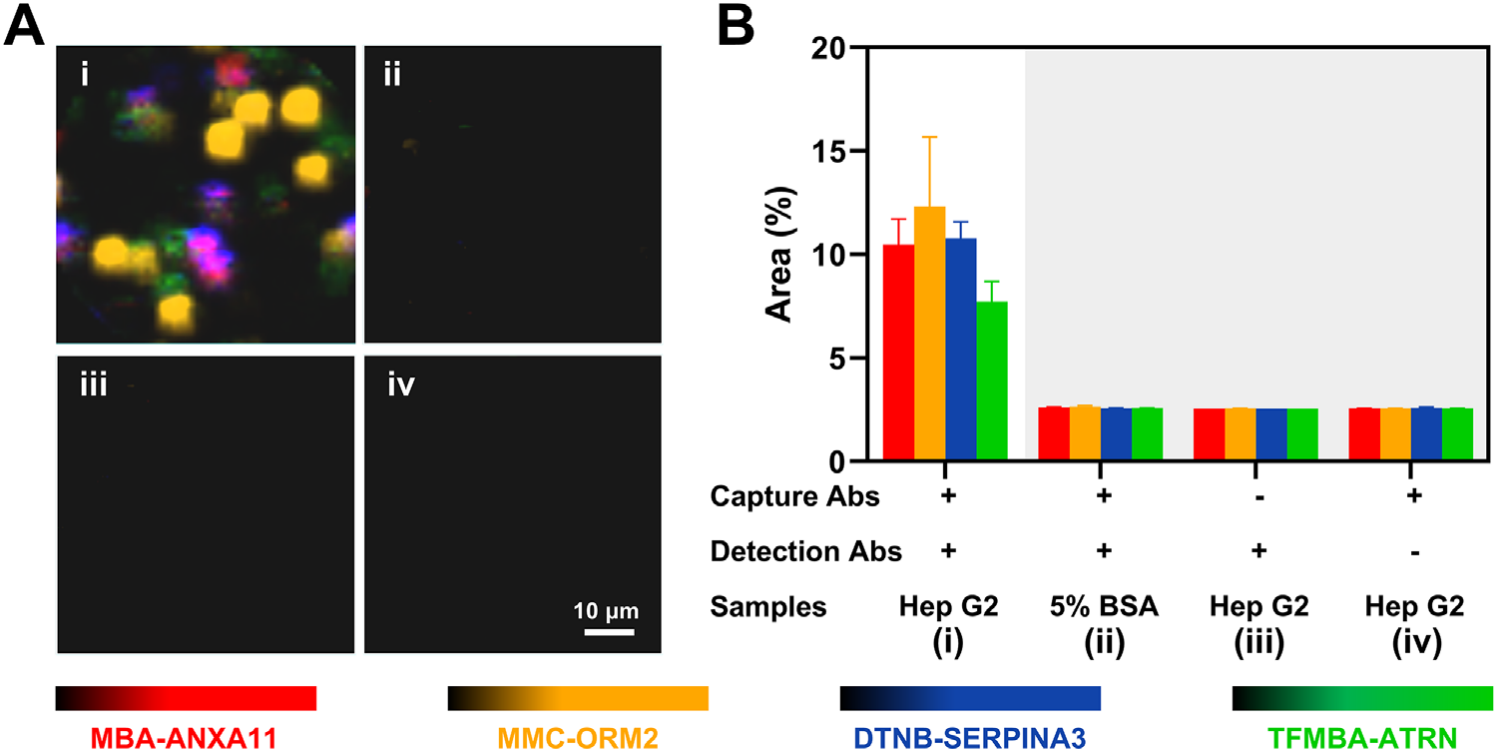
Specificity of the microfluidic‐SERS immunoassay. A) False‐color SERS spectral images were obtained from (i) Hep G2 cell lysate detection, (ii) an assay without cell lysis, (iii) a microfluidic chip lacking capture antibody, and (iv) SERS barcodes without detection antibodies. B) Quantification of the average area of signal‐present pixels. Error bars in Figure 5B indicate the SEM from three independent experiments.

Before analyzing clinical samples, we first tested the nanomixing‐enhanced microfluidic‐SERS immunoassay using cell line lysates, which served as complex biological matrices with reported biomarker expression profiles, to verify assay feasibility under controlled conditions. The Hep G2 cell lysis was strategically chosen (**Figure** **5**A‐(i),B), as this cell line expresses all 4 DEPs, according to the Human Protein Atlas database. Negative controls (Figure 5A (ii—iv),B) included (ii) an assay without cell lysis, (iii) a microfluidic chip without capture antibodies, and (iv) SERS barcodes without detection antibodies. As shown in Figure 5A, characteristic SERS barcode signals were detected in the Hep G2 cell lysis, whereas negative controls exhibited negligible signals, confirming the high specificity of the multiplex microfluidic‐SERS immunoassay for detecting the 4 DEPs in complex biological samples. The enhancement achieved through nanomixing is evident in Figure S1 (Supporting Information), where the signal intensity with nanomixing was up to 2.7 times higher than without nanomixing when detecting lysates from as few as 5000 cells mL^−1^, while signals from cell culture medium controls remained unchanged.

The assay sensitivity was further evaluated by multiplex detection of the 4 target protein biomarkers in Hep G2 cell lysates (50–5×10⁵ cells mL^−1^) in phosphate‐buffered saline (PBS). As shown in Figure S2 (Supporting Information), both the SERS signatures (Figure S2A, Supporting Information) and the signal‐active pixel area of each biomarker (Figure S2B, Supporting Information) increased linearly with the logarithm (base 10) of cell concentration, with squared correlation coefficients (R^2^) ranging from 0.957 to 0.986 within this linear range. The limit of detection (LOD), defined as LOD = 3.3 × (standard error of Y‐intercept)/(slope), was calculated to be 1 cell mL^−1^ for each biomarker (ANXA11, ORM2, SERPINA3, and ATRN). These results confirm the high sensitivity of the developed microfluidic‐SERS platform for detecting low‐concentration biomarkers.

### 2.5 Multiplex Microfluidic‐SERS Immunoassay for Blood Protein Detection

**Figure 6:**
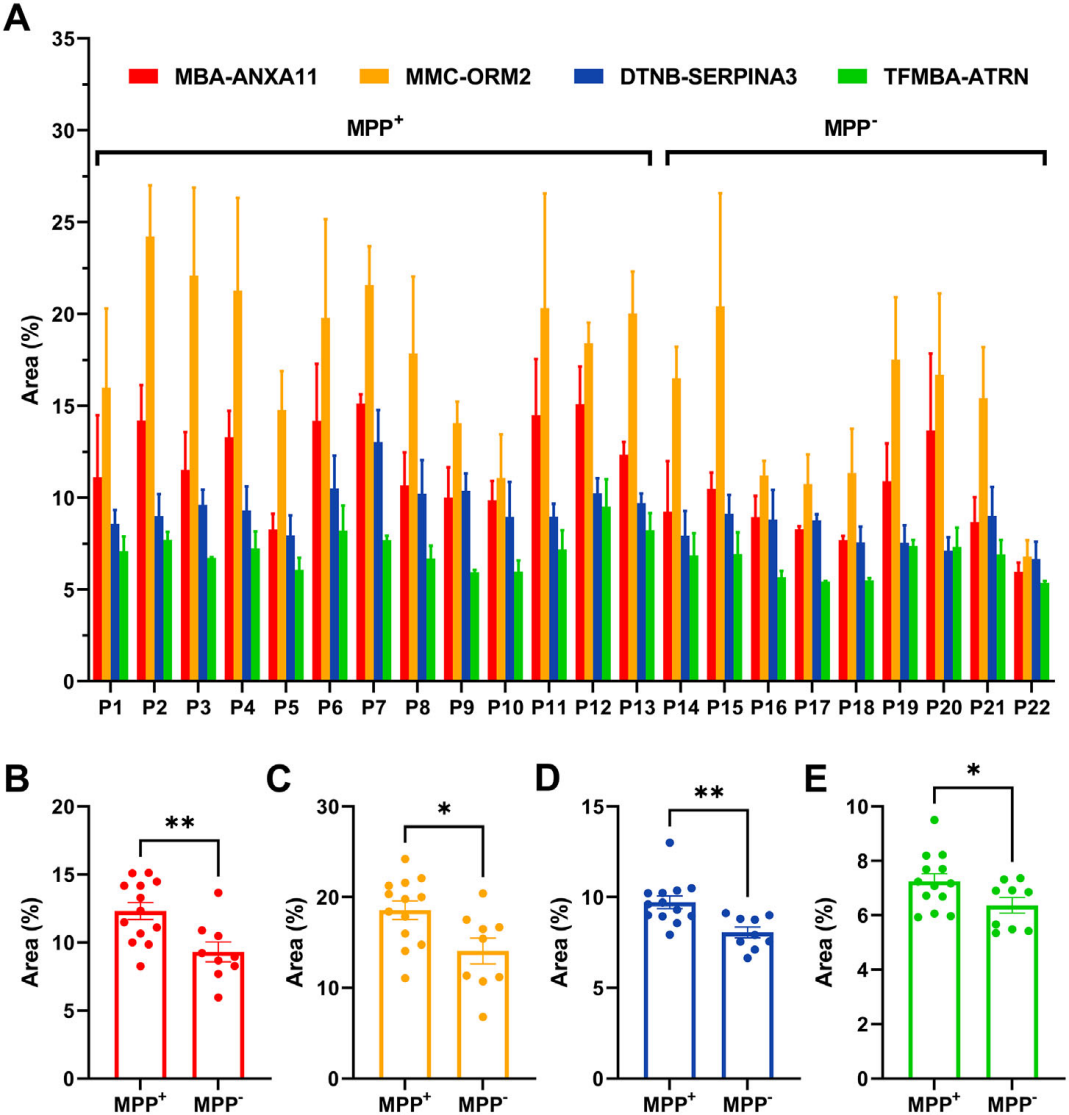
Multiplex SERS detection of ANXA11, ORM2, SERPINA3, and ATRN in serum samples from MPP⁺ (P1‐P13) and MPP⁻ (P14‐P22) LUAD patients. A) SERS signatures of individual patients, with error bars representing the SEM from three independent experiments. Comparison of average expression levels of B) ANXA11, C) ORM2, D) SERPINA3, and E) ATRN between MPP⁺ and MPP⁻ LUAD patients. Statistical significance was assessed using the unpaired two‐tailed t‐test. ^*^ *P* < 0.05; ^**^ *P* < 0.01.

We applied the nanomixing‐integrated microfluidic‐SERS immunoassay to quantify ANXA11, ORM2, SERPINA3, and ATRN expression levels in serum samples from MPP⁺ (P1‐P13) and MPP⁻ LUAD patients (P14‐P22) (**Figure** **6**A). Representative false‐color SERS spectral images of MPP^+^ (P1‐P3) and MPP^−^ (P19‐P21) LUAD patients (Figure S3, Supporting Information) showed a higher number of signal‐active pixels in MPP⁺ LUAD cases. Figure 6B–E compare the expression levels of these 4 DEPs between the two groups, revealing significantly higher levels of all 4 DEPs in MPP⁺ patients compared to MPP⁻ patients. Notably, the SERS immunoassay results were consistent with findings from 4D label‐free quantitative proteomics (Table S1, Supporting Information), further validating the assay's accuracy.

**Figure 7:**
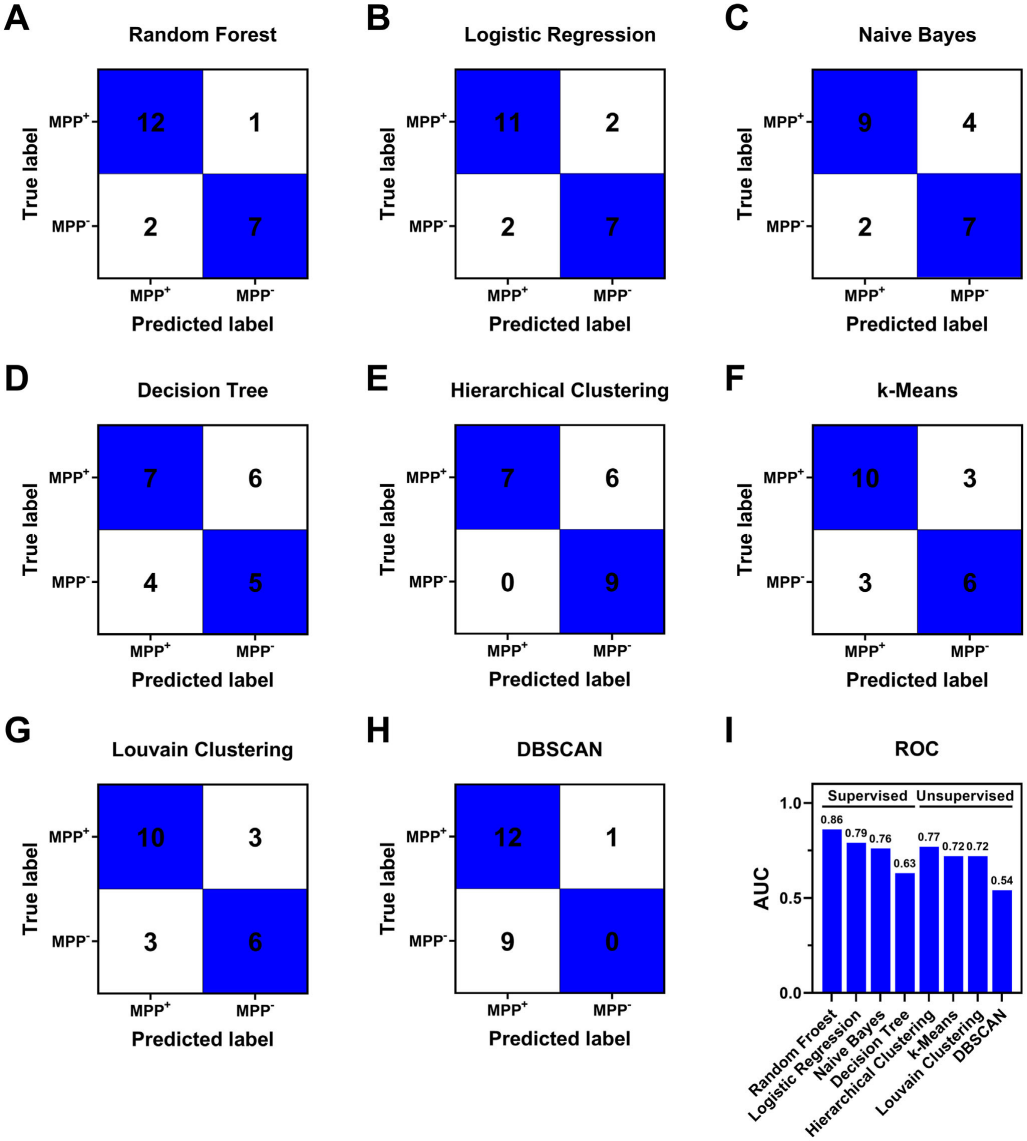
Machine learning models based on SERS signatures of serum ANXA11, ORM2, SERPINA3, and ATRN for differentiating MPP⁺ (P1‐P13) and MPP⁻ (P14‐P22) LUAD patients. A) Random Forest, B) Logistic Regression, C) Naive Bayes, D) Decision Tree, E) Hierarchical Clustering, F) k‐Means, G) Louvain Clustering, H) DBSCAN, and I) their corresponding AUCs.

To evaluate the diagnostic potential of these 4 DEPs in distinguishing MPP⁺ (P1‐P13) and MPP⁻ (P14‐P22) LUAD patients, we applied both supervised and unsupervised machine learning algorithms for a comprehensive classification assessment. Supervised machine learning methods—Random Forest, Logistic Regression, Naive Bayes, and Decision Tree—were applied using labeled datasets to train models for accurate classification between MPP⁺ and MPP⁻ LUAD samples (**Figure 7** A‐D; Figure 4SA‐D, Supporting Information). In parallel, unsupervised machine learning techniques, such as Hierarchical Clustering, k‐Means, Louvain Clustering, and DBSCAN, were used to explore data structure and visualize clustering patterns (Figure 7E‐H; Figure S4E‐H, Supporting Information). All these models are well‐suited for small datasets. Receiver operating characteristic (ROC) analyses were performed to evaluate the performance of these algorithms (Figure 7I; Figure S5, Supporting Information), where values close to 1 indicate perfect performance. The specificity, sensitivity, accuracy, and area under the ROC curve (AUC) values of these models are summarized in Table S2 (Supporting Information).

To further strengthen these findings, we measured these blood protein biomarkers in plasma samples from an independent cohort recruited at a different hospital. This cohort consisted of 10 MPP⁺ LUADs, 10 MPP⁻ LUADs, and 10 healthy controls (Figure S6A, Supporting Information). Similar results were observed, with significantly higher levels of ANXA11 and ORM2 in MPP⁺ LUAD patients compared to MPP⁻ LUAD patients (Figure S6B,C, Supporting Information). Additionally, all 4 biomarkers were significantly elevated in both MPP⁺ and MPP⁻ LUAD patients relative to healthy controls (Figure S6B–E, Supporting Information).

Machine learning analyses were further performed on this dataset, and the results are summarized in Figures S7–S10 and Tables S3 and S4 (Supporting Information). Consistent with our initial findings (Figure 7I; Figure S5, Supporting Information), Random Forest achieved an AUC of 0.81 in distinguishing MPP⁺ from MPP⁻ LUAD patients (Figures S7I and S8, Supporting Information). These values were comparable to or exceeded those reported by Xing et al., who integrated radiomic features, clinical characteristics, and serum tumor biomarkers, achieving AUCs of 0.894 and 0.843 in their training and test cohorts, respectively.^[^ ^5^ ^]^ Additionally, Random Forest, Logistic Regression, and Naïve Bayes yielded AUCs of 0.96, 0.98, and 0.97, respectively, in differentiating LUAD patients from healthy controls (Figures S9I and S10, Supporting Information). Taken together, these findings highlight the potential utility of these blood protein biomarkers for the non‐invasive identification of MPP⁺ LUAD patients.

## 3 Conclusion

Developing a non‐invasive method to predict MPP presence in LUAD patients before surgery is clinically essential, as accurate prediction can guide appropriate resection and treatment decisions. However, differentiating MPP⁺ LUAD from other LUAD subtypes remains highly challenging due to the lack of robust biomarkers and advanced sensing techniques. In particular, the identification of explicit target biomarkers is a prerequisite for applying SERS‐based sensing in disease diagnosis. To address these challenges, we integrated 4D label‐free quantitative proteomics with a microfluidic‐SERS immunoassay, establishing a streamlined workflow from biomarker discovery to detection platform development. We successfully identified DEPs between MPP⁺ and MPP^−^ LUAD patients and elucidated their involvement in key biological processes and pathways. Furthermore, the microfluidic‐SERS immunoassay enabled the detection of identified upregulated DEPs (ANXA11, ORM2, SERPINA3, and ATRN) in blood samples from LUAD patients. Machine learning analyses demonstrated a clear separation between MPP⁺ and MPP^−^ LUAD patients, as well as between LUAD patients and healthy donors. Future studies with larger cohorts are warranted to validate these findings and facilitate clinical translation.

## 4 Experimental Section

### Clinical Sample Acquisition

This study was conducted in accordance with the International Ethical Guidelines for Biomedical Research Involving Human Subjects and the National Statement on Ethical Conduct in Human Research. Ethical approvals were obtained from the Human Research Ethics Committees of Fujian Medical University Union Hospital (2021KY004), Fujian Cancer Hospital (SQ2020‐025–01), and Fujian Normal University. All experiments involving human samples were performed following the approved guidelines, and all patients provided written informed consent. The demographic and clinical data of all participants are summarized in Table S5 (Supporting Information).

### Reagents

All reagents were of analytical grade and used without further purification unless otherwise specified. Chloroauric acid (HAuCl₄) and trisodium citrate were purchased from Sinopharm Chemical Reagent Co., Ltd. MBA, MMC, DTNB, and TFMBA were obtained from Shanghai Macklin Biochemical Co., Ltd. DSP and anti‐ANXA11 antibodies (Cat.: PA5‐96670) were sourced from Thermo Fisher Scientific. Antibodies against ATRN (Cat.: 202440‐T02), ORM2 (Cat.: 13299‐T16), SERPINA3 (Cat.: 10307‐RP02), and recombinant protein A (Cat.: 10600‐P07E) were acquired from Sino Biological. Milli‐Q water (18.2 MΩ cm) was used to prepare all aqueous solutions.

### LC‐MS/MS Analysis

Serum samples were first depleted of high‐abundance proteins. To denature, reduce, and alkylate low‐abundance proteins, the resulting protein fraction was incubated with reaction buffer (1% SDC/100 mm Tris‐HCl, pH 8.5/10 mm Tris(2‐carboxyethyl)phosphine hydrochloride/40 mm 2‐chloroacetamide) at 95 °C for 10 min. The mixture was then diluted with an equal volume of ultrapure water. Protein digestion was carried out by incubating the sample with trypsin at a 1:50 mass ratio (enzyme to protein) at 37 °C overnight. The reaction was quenched by adding trifluoroacetic acid, followed by centrifugation at 16000× g to remove the supernatant. Peptides were then desalted using an in‐house‐made styrene‐divinylbenzene column.

LC‐MS/MS analysis was performed using an UltiMate 3000 RSLCnano system (Thermo) coupled to a hybrid trapped ion mobility‐quadrupole time‐of‐flight mass spectrometer (timsTOF Pro, Bruker Daltonics, Bremen, Germany) via a CaptiveSpray ion source. Digested proteome mixtures were first trapped on a C18 HPLC column (75 µm × 2 cm, 3 µm particle size, 100 Å pore size, Thermo) and subsequently separated using a reversed‐phase C18 HPLC column (75 µm × 25 cm, 1.6 µm particle size, 100 Å pore size, IonOpticks) with an autosampler. A binary gradient was established using mobile phase A (0.1% v/v formic acid) and mobile phase B (0.1% v/v formic acid, 99.9% v/v acetonitrile) at a flow rate of 300 nL min^−1^.

The mass spectrometer was operated in parallel accumulation‐serial fragmentation (PASEF) mode with a capillary voltage of 1500 V. MS1 and MS2 scans were acquired over an m/z range of 100–1700. The ion mobility 1/K₀ range was set from 0.75 to 1.4 V s cm^−2^. Both the accumulation and ramp times were set to 100 ms. The total acquisition cycle of 1.16 s consisted of one MS1 full scan and ten MS2 scans. Singly charged precursor ions were excluded. Precursors for MS/MS were selected at an intensity threshold of 1000 arbitrary units (a.u.) and re‐sequenced until reaching a target intensity of 10000 a.u., with a dynamic exclusion window of 40 s. The quadrupole isolation window was set to 2.0 Da for m/z 700 and 3.0 Da for m/z 800. Collision energy was linearly reduced from 59 eV at 1/K₀ = 1.6 V s cm^−2^ to 20 eV at 1/K₀ = 0.6 V s cm^−2^ based on ion mobility settings.

Acquired spectra were searched against the human proteome Uniprot database (released June 19, 2023, with 20423 entries) using MaxQuant software (V2.0.1). Oxidation of methionine and N‐terminal acetylation were set as variable modifications, while carbamidomethylation was set as a fixed modification. Trypsin was specified as the proteolytic enzyme. The initial precursor mass tolerances were set to 20 ppm in the first search and 4.5 ppm in the main search, with a fragment mass tolerance of 20 ppm. The false discovery rate was set to 1% at both the peptide spectral match and protein levels, and peptide identification was enabled for matching between runs.

### Bioinformatics Analysis

DEP analysis was performed to identify proteins with altered expression between LUAD samples with and without MPP patterns. To investigate the potential roles of these DEPs in the pathogenesis of LUAD with MPP patterns, enrichment analyses, including GO annotation and KEGG pathway analysis, were conducted. Additionally, a protein interaction network was constructed using the STRING database to explore the relationships among DEPs.

### Synthesis of AuNPs and SERS Barcodes

AuNPs with a 60‐nm diameter were synthesized via citrate reduction of HAuCl₄ following a previously reported method.^[^ ^23^ ^]^ AuNPs (1 mL) were incubated with 10 µL of 1 mm Raman reporters (MBA, MMC, DTNB, or TFMBA in ethanol) and 2 µL of 1 mm DSP in dimethyl sulfoxide. After incubation, the mixture was centrifuged and resuspended in 200 µL of 0.1 mm PBS. Protein A (0.5 µg) was then added to react with the NHS esters of DSP, forming covalent amide bonds to enable subsequent conjugation of 0.5 µg polyclonal antibodies. The resulting antibody‐functionalized SERS barcodes were resuspended in 200 µL of 0.1% (w/v) bovine serum albumin (BSA) for blocking and stored at 4 °C.

### Nanoparticle Characterization

Transmission electron microscopy images of AuNPs were acquired using an FEI Talos 200S operated at 200 kV. Scanning electron microscopy images were obtained with a ZEISS GeminiSEM 300 at 3 kV. UV–vis extinction spectra of AuNPs were recorded using a PerkinElmer Lambda 950 UV–vis spectrometer. Particle size distribution and zeta potential were measured using a ZetaView PMX‐x30.

### Microfluidic Chip Fabrication and Functionalization

The microfluidic chip was designed and fabricated as described in the previous publications.^[^ ^13^, ^24^ ^]^ The chip consisted of a glass substrate with arrays of gold electrodes structured asymmetrically—an inner circular electrode (diameter: 1000 µm) and an outer ring‐shaped electrode (width: 120 µm), separated by 900 µm. The chip was fabricated via photolithography following a previously reported method.^[^ ^13^ ^]^ Briefly, the electrode pattern was designed and transferred onto a 5‐inch chrome mask using a direct laser writer. A 4‐inch high‐boron glass wafer was spin‐coated with NR7‐1500PY at 1200 rpm for 10 s and 3000 rpm for 40 s, then baked at 150 °C for 1 min. UV exposure was performed using a SUSS MICROTEC system, followed by a second bake at 120 °C for 2 min. The chip was developed in tetramethylammonium hydroxide for 7 s to reveal the negative electrode structure. The developed wafer was coated with a 10‐nm chromium layer followed by 200‐nm gold deposition. The final electrode structure was obtained through overnight lift‐off in tetramethylammonium hydroxide.

A polydimethylsiloxane (PDMS) microwell array was fabricated by curing activated silicone elastomer (SYLGARD 184) in a vacuum drying oven at 80 °C for 30 min, then punching microwell arrays (diameter: 6 mm) aligned to the gold electrodes. The PDMS microwell array was thermally bonded to the chip at 80 °C for 4 h, completing the device assembly.

After binding the PDMS microwell array to the chip, 10 µL of 5 mm DSP in dimethyl sulfoxide was added to each well and incubated for 2 h, followed by sequential washing—once with ethanol and three times with PBS. Recombinant protein A (10 µL, 10 µg mL⁻¹ in PBS) was then introduced and incubated for 1 h. After a PBS wash, the gold electrodes were incubated with 10 µL of 5 µg mL⁻¹ antibodies against ANXA11, ORM2, SERPINA3, ATRN and for 2 h, followed by blocking with 5% (w/v) BSA for another 2 h. All steps were performed at room temperature. Before use, the microwells were rinsed with PBS.

### Nanomixing‐Enhanced Microfluidic Assay

In each microfluidic well, 50 µL of sample was loaded, and an 800‐mV field at 500 Hz was applied for 30 min. The wells were then washed three times with BSA‐PBS buffer (1% w/v BSA in PBS) to remove unbound components. Next, 25 µL of SERS barcodes prepared in BSA‐PBS buffer were introduced and subjected to the same field conditions for 45 min. To further eliminate nonspecific binding, another triple wash with BSA‐PBS buffer was performed. After washing, all remaining liquid was completely removed from the wells.

### SERS Measurements

SERS signals were measured using a confocal HORIBA Raman spectrometer (XploRA PLUS) equipped with a 785‐nm laser, a 1200 g mm⁻¹ grating blazed at 750 nm, a 300‐µm confocal hole, a 100‐µm entrance, and an electron‐multiplying charge‐coupled device. The spectrometer was calibrated using the characteristic silicon wafer peak at 520 cm⁻¹ before measurements.

For SERS detection, gold electrodes underwent spectral mapping over a 60 µm × 60 µm area (60 × 60 pixels) using a 50× long‐working‐distance microscope objective (×50_VIS_LWD). Each spectrum (400–1800 cm⁻¹) was acquired with 0.05 s of irradiation at 38 mW laser power. SERS spectral mapping images were generated based on the characteristic peak intensities of antibody‐conjugated SERS barcodes: 1075 cm⁻¹ (MBA), 1175 cm⁻¹ (MMC), 1337 cm⁻¹ (DTNB), and 1375 cm⁻¹ (TFMBA).

### Machine Learning Analysis

Random Forest, Logistic Regression, Naive Bayes, Decision Tree, Hierarchical Clustering, k‐Means, Louvain Clustering, and DBSCAN algorithms were applied using Orange Data Mining (Version 3.34) with the following parameter settings: A) Random Forest: the minimum size of split subsets was set to 4; B) Logistic Regression: L2 regularization was used, with a regularization strength of 3; C) Naive Bayes: applied with default automatic settings; D) Decision Tree: a binary tree was built with a minimum of 5 instances in leaves, splitting was stopped when subsets contained fewer than 5 instances, the maximum tree depth was set to 50, and tree growth stopped when majority purity reached 95%; E) Hierarchical Clustering: performed using Ward linkage to measure distances between clusters, with the top two clusters selected for analysis; F) k‐Means: column normalization was applied, and the number of clusters was fixed at 2. Random initialization was used for cluster centroids, with a maximum of 300 iterations and 10 restarts; G) Louvain Clustering: data normalization was performed, and principal component analysis was applied to reduce noise. The Manhattan distance metric and 21 nearest neighbors were used to construct the graph, with the resolution parameter set at 0.9; H) DBSCAN: a core point was defined as having at least one neighbor within a maximum distance (epsilon) of 1.74. The Euclidean distance metric was used, and data were standardized column‐wise.

All learning results were visualized using confusion matrices, ROC curves, and 2D projections to enable intuitive interpretation of class separations and model performance.

### Statistical Analysis

All SERS spectra were background‐corrected using LabSpec 6 software. The area proportion of signal‐active pixels was quantified using ImageJ, normalized to the total mapping area, and used to calculate the SERS signature. Data are presented as mean ± SEM. Unpaired two‐sided t‐tests and one‐way ANOVA (with an alpha value of 0.05) were performed using GraphPad Prism (version 10) to compare two and three independent groups, respectively.

## Conflict of Interest

The authors declare no conflict of interest.

## Supporting information



Supporting Information

## Data Availability

The data that support the findings of this study are available from the corresponding author upon reasonable request.
