# Supplementary material for: Proteomics‐Empowered Microfluidic‐SERS Immunoassay for Identifying and Detecting Biomarkers of Micropapillary Lung Adenocarcinoma
Source: Adv Sci (Weinh). 2025 May 3;12(25):2501336. doi: 10.1002/advs.202501336 (PMC12224922; doi:10.1002/advs.202501336)
Supplement: Supplementary file 1 — Supporting Information [file ADVS-12-2501336-s001.docx]

**Supporting Information**

**Proteomics-empowered Microfluidic-SERS Immunoassay for Identifying and Detecting Biomarkers of Micropapillary Lung Adenocarcinoma**

Dechun Zhang^1,^†, Kaiming Peng^2,^†, Hui Xu^2^, Yanping Chen^3^, Jing Wang^1,^*

^1^Key Laboratory of OptoElectronic Science and Technology for Medicine of Ministry of Education, Fujian Provincial Key Laboratory of Photonics Technology, Fujian Normal University, Fuzhou 350117, Fujian, China

^2^Department of Thoracic Surgery, Fujian Medical University Union Hospital, Fuzhou 350001, Fujian, China

^3^Department of Pathology, Clinical Oncology School of Fujian Medical University and Fujian Cancer Hospital, Fuzhou, Fujian 350014, China

†Equal contribution.

*Corresponding author: jing.wang@fjnu.edu.cn


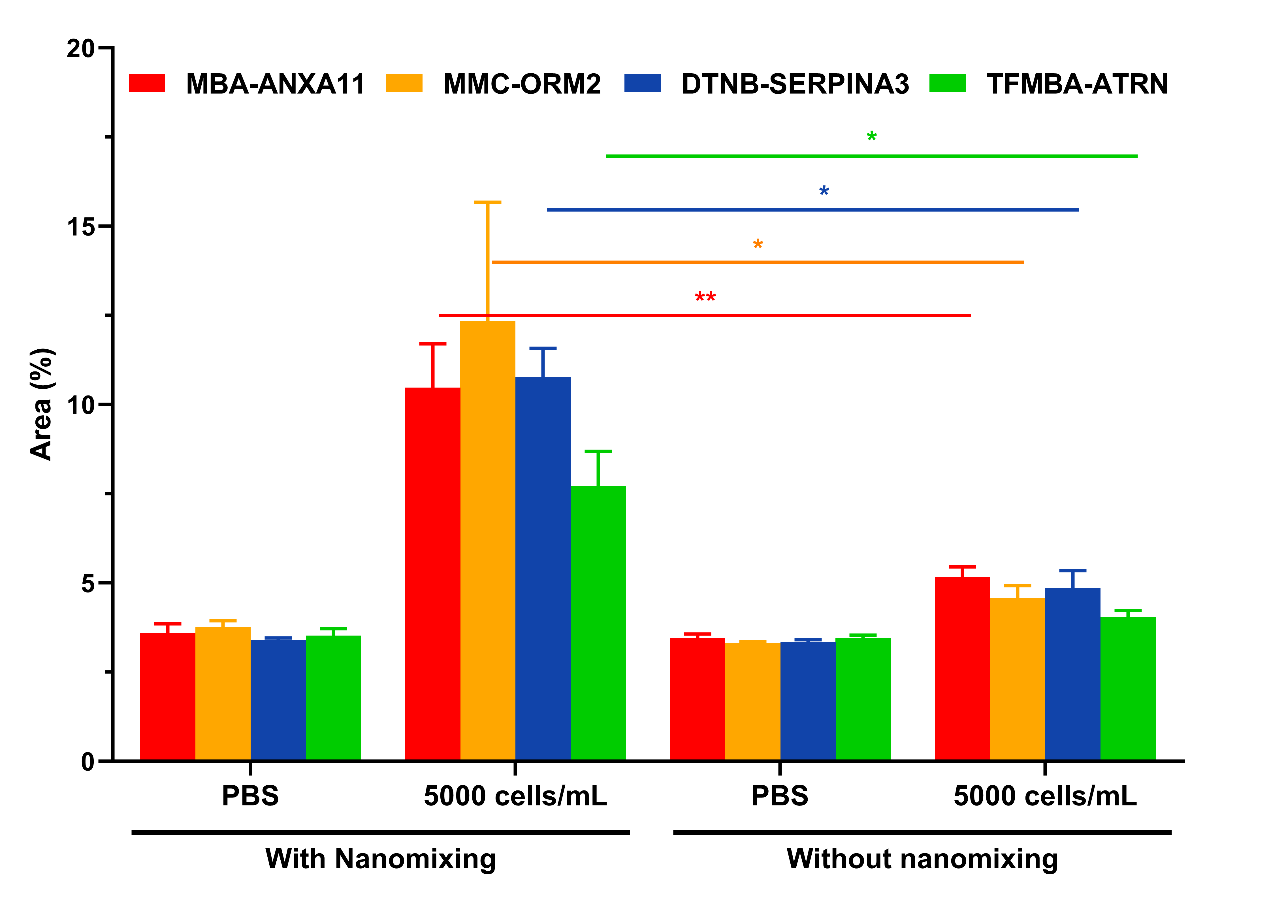
Figure S1. Assay performance comparison with and without nanomixing. Statistical significance was assessed using the unpaired two-tailed t-test. **P* < 0.05; ***P* < 0.01.


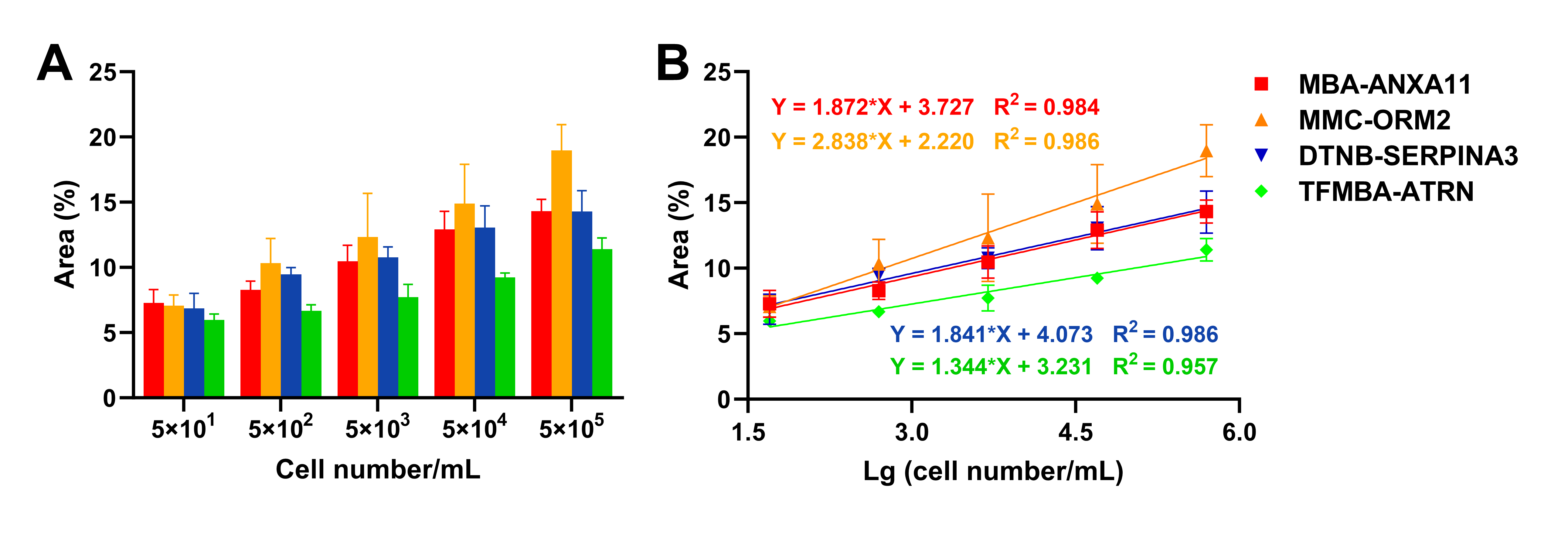
Figure S2. Assay sensitivity in the multiplex detection of four target protein biomarkers in Hep G2 cell lysates (50–5×10⁵ cells/mL) in PBS. (A) Histogram showing the increase in SERS signatures with the logarithm (base 10) of cell concentration. (B) Linear regression curves showing the relationship between the logarithm (base 10) of cell concentration and the signal-active pixel area of each biomarker. Error bars represent the SEM from three independent experiments.


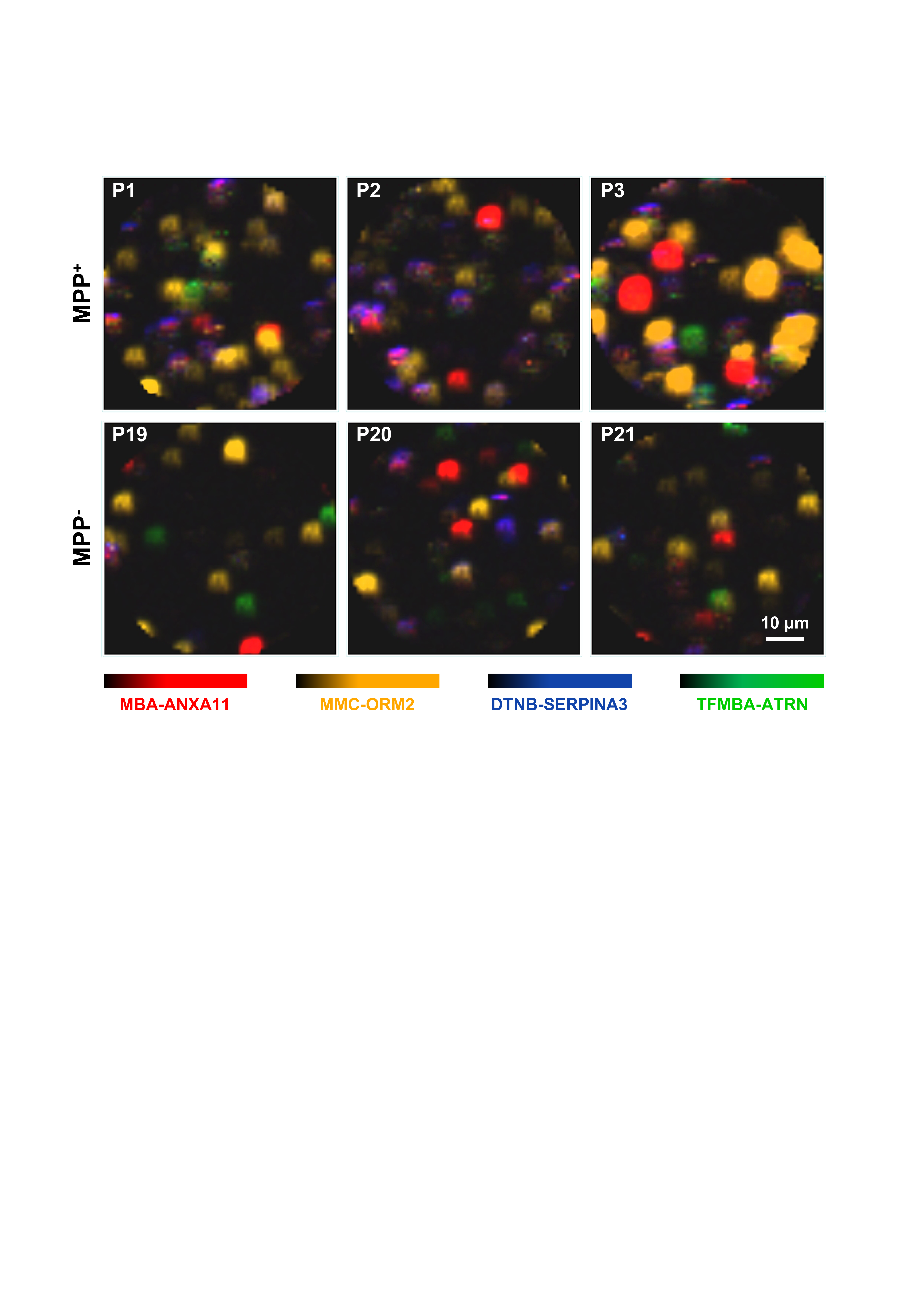


Figure S3. Representative false-color SERS spectral images of MPP^+^ (P1, P2, and P3) and MPP^-^ (P19, P20, and P21) patients.


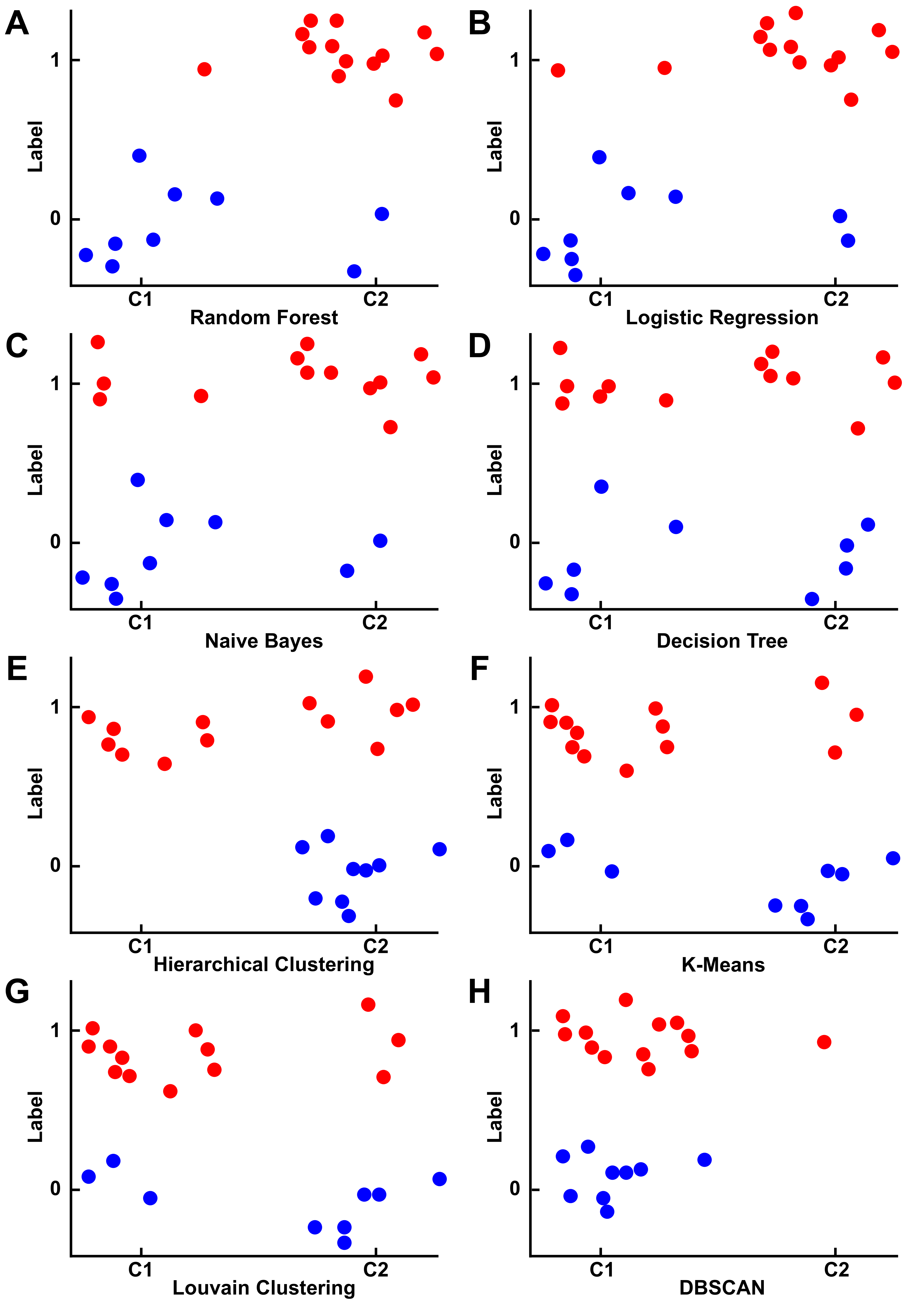


Figure S4. Scatter plots comparing true categories with clustering results (C1 *vs.* C2) for differentiating MPP⁺ (P1-P13) and MPP⁻ (P14-P22) LUAD patients, using supervised and unsupervised machine learning algorithms. (A) Random Forest, (B) Logistic Regression, (C) Naive Bayes, (D) Decision Tree, (E) Hierarchical Clustering, (F) k-Means, (G) Louvain Clustering, and (H) DBSCAN.


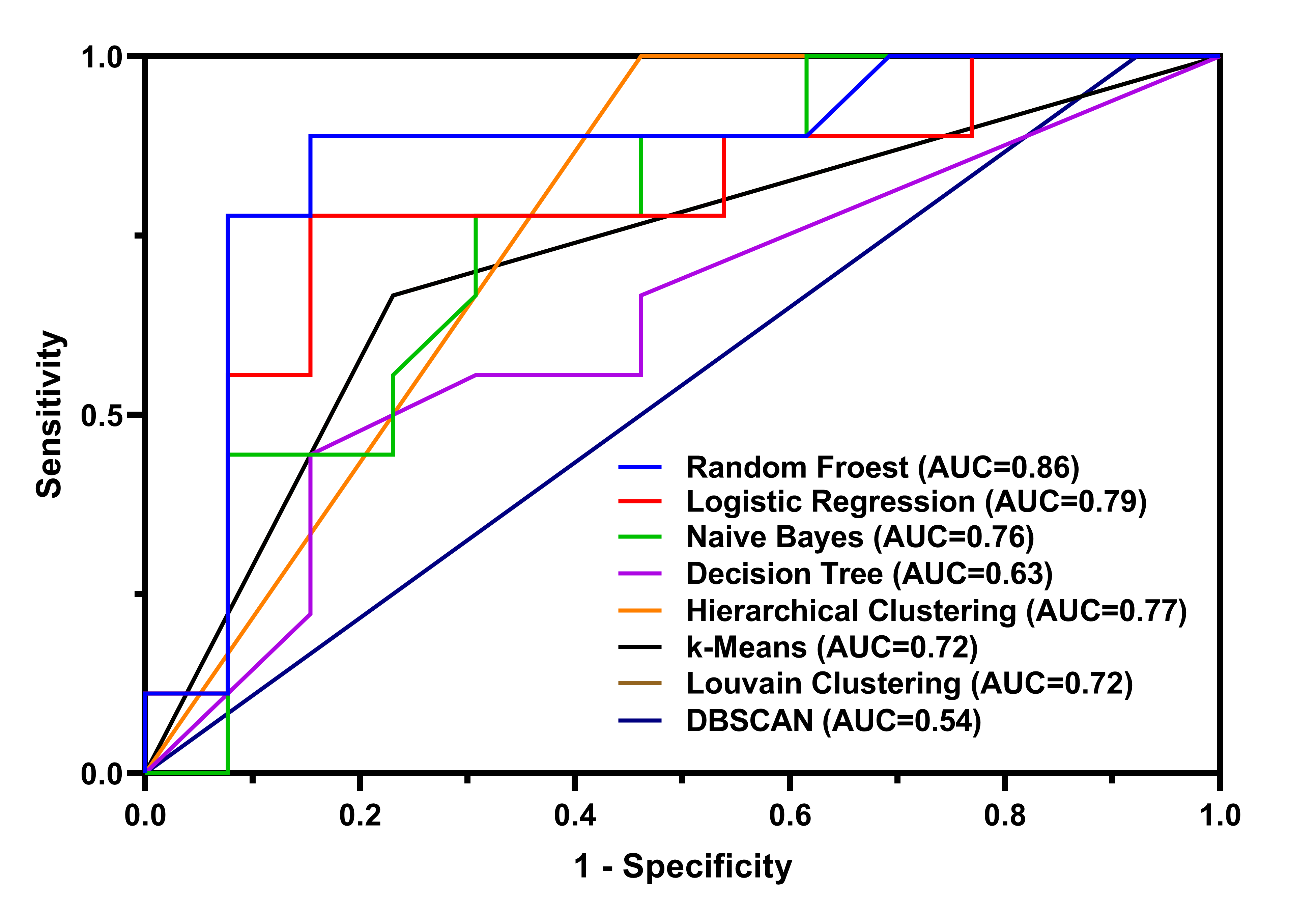


Figure S5. ROC curves evaluating the performance of machine learning algorithms in differentiating MPP⁺ (P1-P13) and MPP⁻ (P14-P22) LUAD patients.


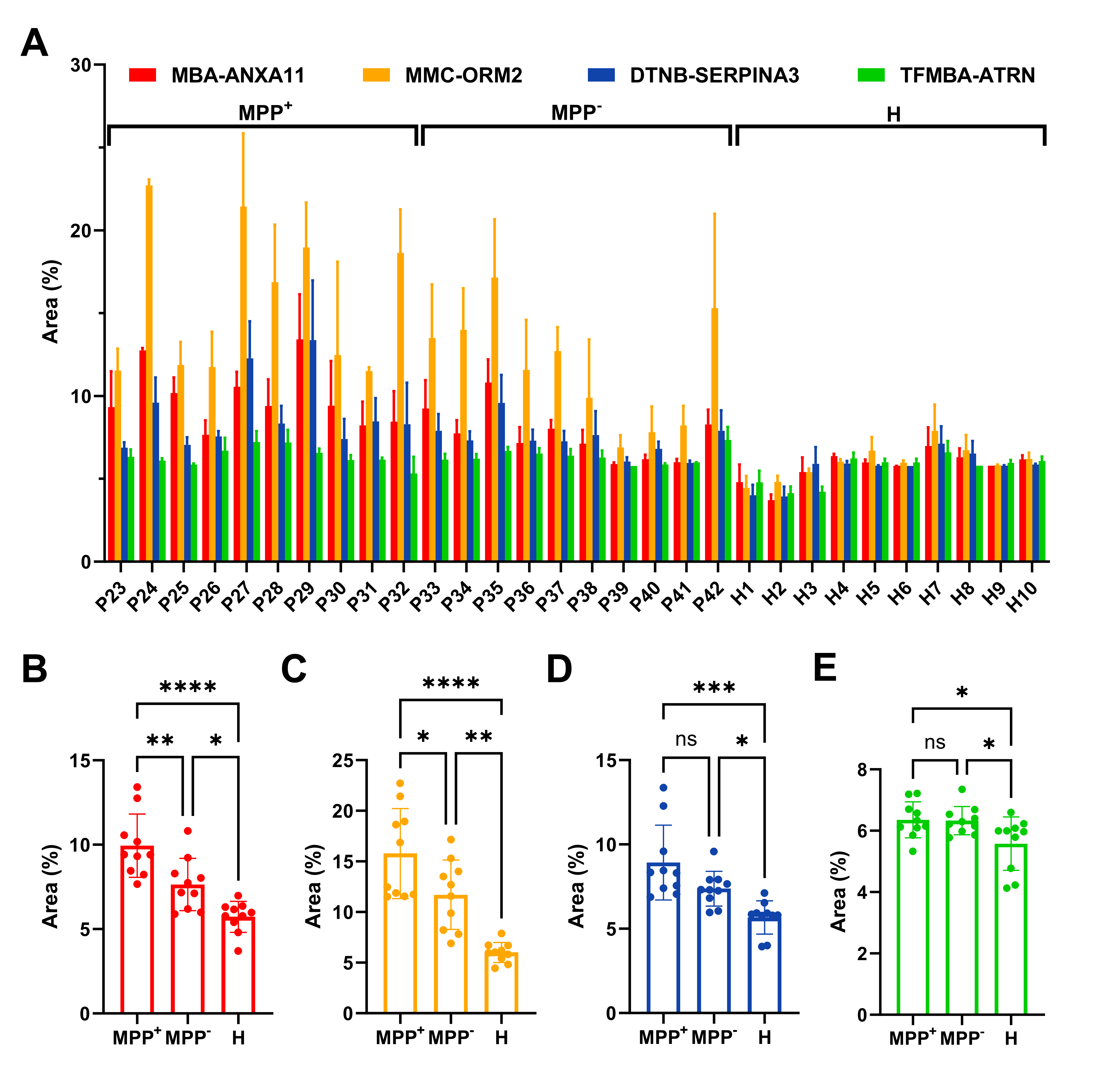
Figure S6. Multiplex SERS detection of ANXA11, ORM2, SERPINA3, and ATRN in plasma samples from MPP⁺ (P23-P32) and MPP⁻ (P33-P42) LUAD patients, as well healthy donors (H1-H10). (A) SERS signatures of individuals, with error bars representing the SEM from three independent experiments. Comparison of average expression levels of (B) ANXA11, (C) ORM2, (D) SERPINA3, and (E) ATRN among MPP⁺ and MPP⁻ LUAD patients and healthy donors. Statistical significance was assessed using one-way ANOVA with Tukey’s multiple comparison correction. ns, not significant; **P* < 0.0332; ***P* < 0.0021; ****P* < 0.0002; *****P* < 0.0001.


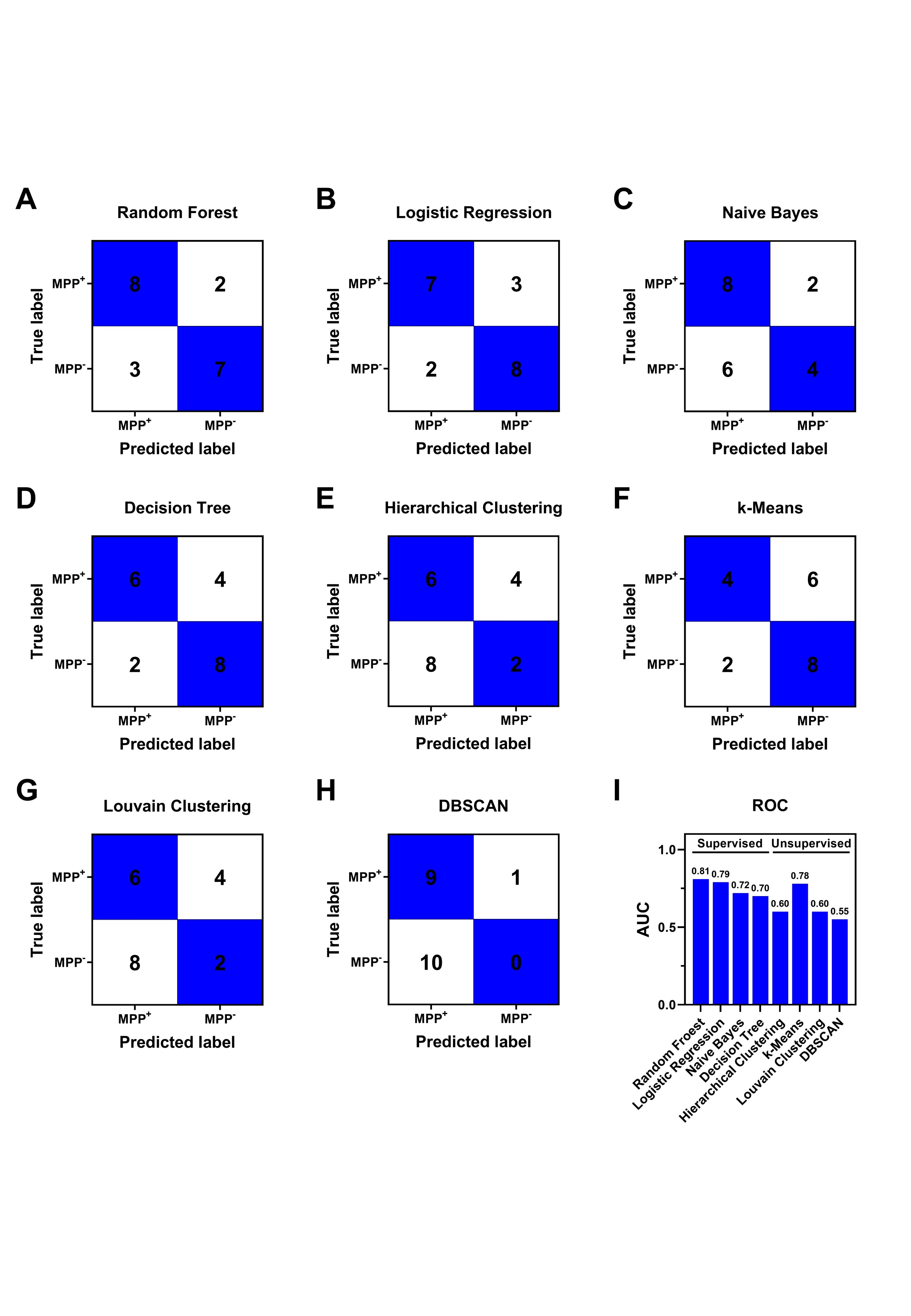


Figure S7. Machine learning models based on SERS signatures of plasma ANXA11, ORM2, SERPINA3, and ATRN for differentiating MPP⁺ (P23-P32) and MPP⁻ (P33-P42) LUAD patients. (A) Random Forest, (B) Logistic Regression, (C) Naive Bayes, (D) Decision Tree, (E) Hierarchical Clustering, (F) k-Means, (G) Louvain Clustering, (H) DBSCAN, and (I) their corresponding AUCs.


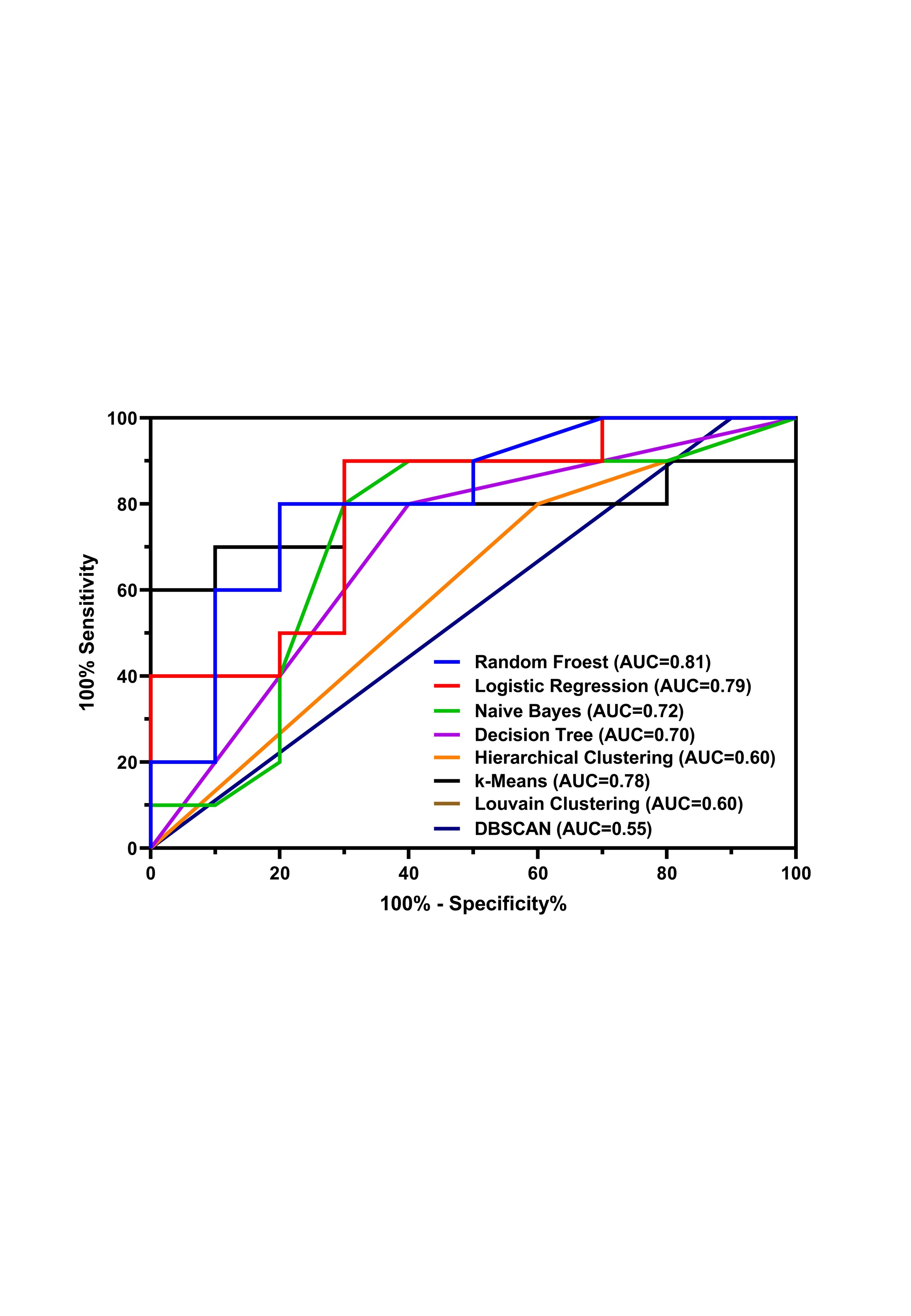


Figure S8. ROC curves evaluating the performance of machine learning algorithms in differentiating MPP⁺ (P23-P32) and MPP⁻ (P33-P42) LUAD patients.


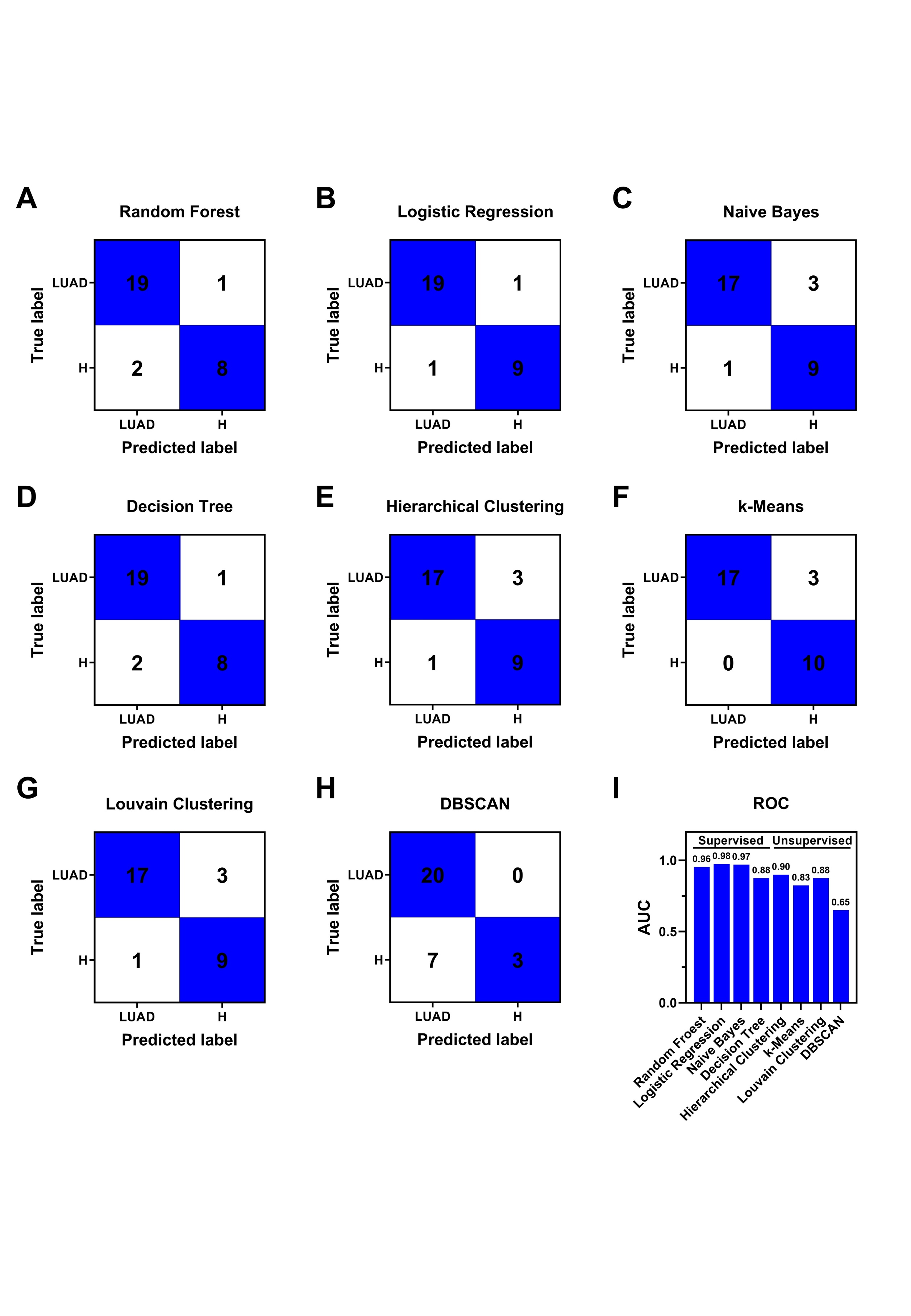


Figure S9. Machine learning models based on SERS signatures of plasma ANXA11, ORM2, SERPINA3, and ATRN for differentiating LUAD patients (P23-P42) and healthy donors (H1-H10). (A) Random Forest, (B) Logistic Regression, (C) Naive Bayes, (D) Decision Tree, (E) Hierarchical Clustering, (F) k-Means, (G) Louvain Clustering, (H) DBSCAN, and (I) their corresponding AUCs.


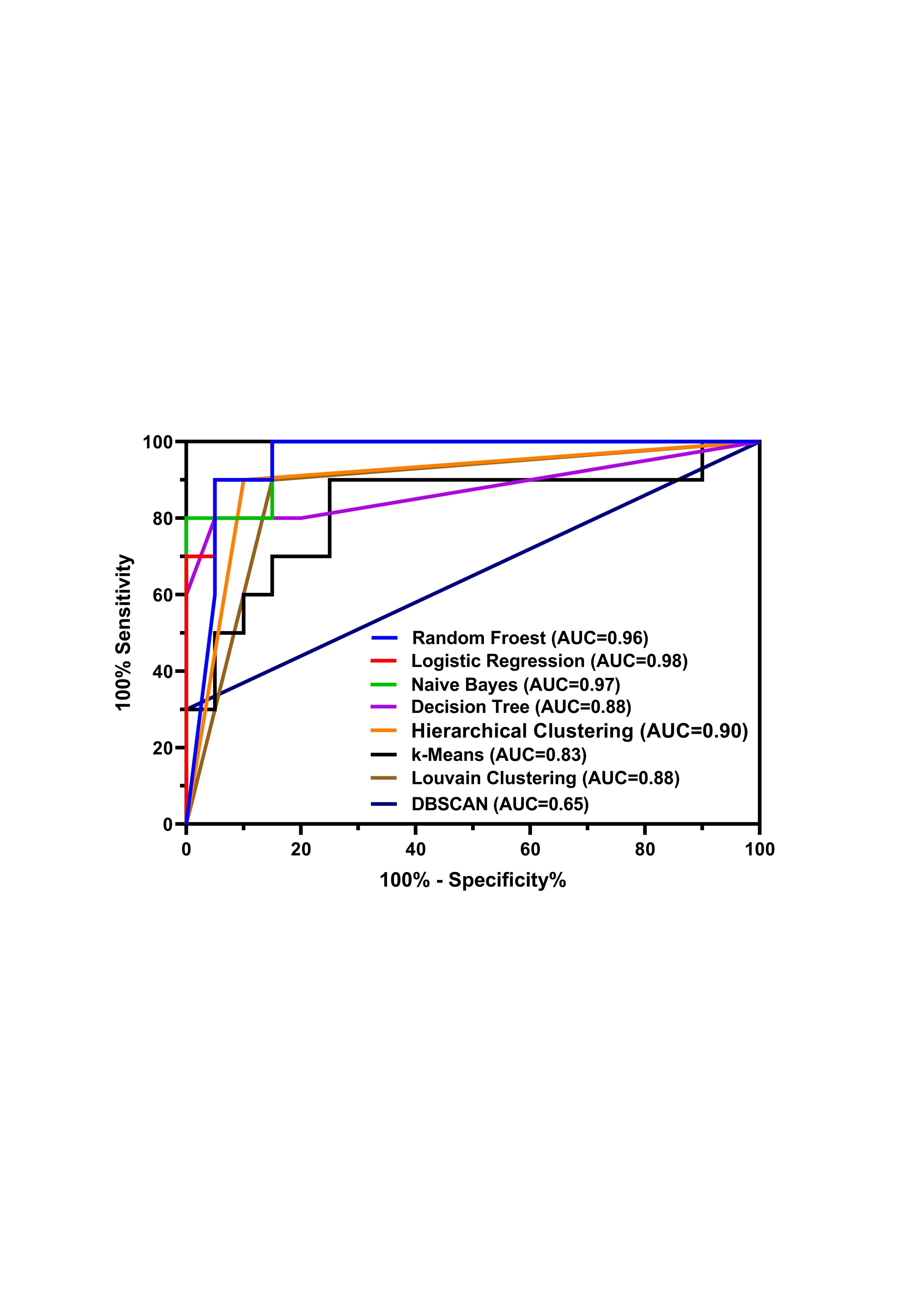


Figure S10. ROC curves evaluating the performance of machine learning algorithms in differentiating LUAD patients (P23-P42) and healthy donors (H1-H10).

Table S1 Demographic characteristics of clinical samples

| Sample | Age | Gender | Micropapillary component % | Serum/Plasma |
| --- | --- | --- | --- | --- |
| P1 | 45 | Male | 40 | Serum |
| P2 | 56 | Female | 10 | Serum |
| P3 | 56 | Female | 5 | Serum |
| P4 | 61 | Female | 5 | Serum |
| P5 | 77 | Male | ≤5 | Serum |
| P6 | 77 | Male | 10 | Serum |
| P7 | 55 | Male | 10 | Serum |
| P8 | 53 | Male | 20 | Serum |
| P9 | 66 | Male | ≤5 | Serum |
| P10 | 50 | Female | 5 | Serum |
| P11 | 58 | Male | 5 | Serum |
| P12 | 74 | Male | 10 | Serum |
| P13 | 52 | Male | 10 | Serum |
| P14 | 77 | Male | 0 | Serum |
| P15 | 34 | Female | 0 | Serum |
| P16 | 56 | Female | 0 | Serum |
| P17 | 73 | Female | 0 | Serum |
| P18 | 62 | Male | 0 | Serum |
| P19 | 67 | Male | 0 | Serum |
| P20 | 48 | Female | 0 | Serum |
| P21 | 65 | Female | 0 | Serum |
| P22 | 60 | Female | 0 | Serum |
| P23 | 75 | Female | ≤5 | Plasma |
| P24 | 58 | Female | 10 | Plasma |
| P25 | 72 | Female | ≤5 | Plasma |
| P26 | 74 | Male | 10 | Plasma |
| P27 | 64 | Female | 35 | Plasma |
| P28 | 49 | Female | 25 | Plasma |
| P29 | 64 | Female | 5 | Plasma |
| P30 | 60 | Male | ≤5 | Plasma |
| P31 | 66 | Male | 10 | Plasma |
| P32 | 61 | Female | 10 | Plasma |
| P33 | 65 | Male | 0 | Plasma |
| P34 | 56 | Female | 0 | Plasma |
| P35 | 54 | Male | 0 | Plasma |
| P36 | 61 | Male | 0 | Plasma |
| P37 | 40 | Male | 0 | Plasma |
| P38 | 58 | Female | 0 | Plasma |
| P39 | 67 | Male | 0 | Plasma |
| P40 | 55 | Male | 0 | Plasma |
| P41 | 48 | Male | 0 | Plasma |
| P42 | 57 | Male | 0 | Plasma |
| H1 | 78 | Male | 0 | Plasma |
| H2 | 59 | Male | 0 | Plasma |
| H3 | 61 | Female | 0 | Plasma |
| H4 | 69 | Female | 0 | Plasma |
| H5 | 68 | Male | 0 | Plasma |
| H6 | 45 | Female | 0 | Plasma |
| H7 | 22 | Male | 0 | Plasma |
| H8 | 59 | Male | 0 | Plasma |
| H9 | 46 | Male | 0 | Plasma |
| H10 | 56 | Female | 0 | Plasma |

Table S2 Differentially expressed proteins identified by 4D label-free proteomics

| Accession | Proteins | Up / down-regulation |
| --- | --- | --- |
| A0A0C4DH31 | IGHV1-18 | Up |
| O75882 | ATRN | Up |
| P01011 | SERPINA3 | Up |
| P19652 | ORM2 | Up |
| P50995 | ANXA11 | Up |
| Q13790 | APOF | Up |
| Q7Z4R8 | C6orf120 | Up |
| O14672 | ADAM10 | Down |
| P01889 | HLA-B | Down |
| P07093 | SERPINE2 | Down |
| P07585 | DCN | Down |
| P13489 | RNH1 | Down |
| P13497 | BMP1 | Down |
| P19086 | GNAZ | Down |
| P19971 | TYMP | Down |
| P22314 | UBA1 | Down |
| P29350 | PTPN6 | Down |
| P29508 | SERPINB3 | Down |
| P30041 | PRDX6 | Down |
| P40306 | PSMB10 | Down |
| P48059 | LIMS1 | Down |
| P49746 | THBS3 | Down |
| P56202 | CTSW | Down |
| P61769 | B2M | Down |
| Q12797 | ASPH | Down |
| Q13418 | ILK | Down |
| Q15019 | SEPTIN2 | Down |
| Q3ZCW2 | LGALSL | Down |
| Q6IBS0 | TWF2 | Down |
| Q6V0I7 | FAT4 | Down |
| Q71U36 | TUBA1A | Down |
| Q86UX7 | FERMT3 | Down |
| Q92954 | PRG4 | Down |
| Q96AX2 | RAB37 | Down |
| Q9H4B7 | TUBB1 | Down |
| Q9HBI1 | PARVB | Down |
| Q9NRA1 | PDGFC | Down |
| Q9NY65 | TUBA8 | Down |
| Q9NYQ6 | CELSR1 | Down |
| P55157 | MTTP | Down |
| P63267 | ACTG2 | Down |
| Q14956 | GPNMB | Down |
| P35052 | GPC1 | Down |
| O95084 | PRSS23 | Down |

Table S3 Diagnostic performance of the microfluidic-SERS platform in differentiating MPP⁺ (P1-P13) and MPP⁻ (P14-P22) LUAD patients

| Diagnostic model | Specificity | Sensitivity | Accuracy | AUC |
| --- | --- | --- | --- | --- |
| Random Forest | 0.78 | 0.92 | 0.86 | 0.86 |
| Logistic Regression | 0.78 | 0.85 | 0.82 | 0.79 |
| Naive Bayes | 0.78 | 0.69 | 0.73 | 0.76 |
| Decision Tree | 0.56 | 0.54 | 0.55 | 0.63 |
| Hierarchical Clustering | 1.00 | 0.54 | 0.73 | 0.77 |
| k-Means | 0.67 | 0.77 | 0.73 | 0.72 |
| Louvain Clustering | 0.67 | 0.77 | 0.73 | 0.72 |
| DBSCAN | 0.00 | 0.92 | 0.54 | 0.54 |

Table S4 Diagnostic performance of the microfluidic-SERS platform in differentiating MPP⁺ (P23-P32) and MPP⁻ (P33-P42) LUAD patients

| Diagnostic model | Specificity | Sensitivity | Accuracy | AUC |
| --- | --- | --- | --- | --- |
| Random Forest | 0.70 | 0.80 | 0.75 | 0.81 |
| Logistic Regression | 0.80 | 0.70 | 0.75 | 0.79 |
| Naive Bayes | 0.40 | 0.80 | 0.60 | 0.72 |
| Decision Tree | 0.80 | 0.60 | 0.70 | 0.70 |
| Hierarchical Clustering | 0.20 | 0.60 | 0.40 | 0.60 |
| k-Means | 0.80 | 0.40 | 0.60 | 0.78 |
| Louvain Clustering | 0.20 | 0.60 | 0.40 | 0.60 |
| DBSCAN | 0.00 | 0.90 | 0.45 | 0.55 |
|  |  |  |  |  |

Table S5 Diagnostic performance of the microfluidic-SERS platform in differentiating LUAD patients (P23-P42) and healthy donors (H1-H10)

| Diagnostic model | Specificity | Sensitivity | Accuracy | AUC |
| --- | --- | --- | --- | --- |
| Random Forest | 0.80 | 0.95 | 0.90 | 0.96 |
| Logistic Regression | 0.90 | 0.95 | 0.93 | 0.98 |
| Naive Bayes | 0.90 | 0.85 | 0.87 | 0.97 |
| Decision Tree | 0.80 | 0.95 | 0.90 | 0.88 |
| Hierarchical Clustering | 0.90 | 0.85 | 0.87 | 0.90 |
| k-Means | 1.00 | 0.85 | 0.90 | 0.83 |
| Louvain Clustering | 0.90 | 0.85 | 0.87 | 0.88 |
| DBSCAN | 0.30 | 1.00 | 0.77 | 0.65 |
